# Impact of roe enhancement on quality parameters in sea urchins *Echinus esculentus* and *Strongylocentrotus droebachiensis*

**DOI:** 10.1038/s41538-025-00579-5

**Published:** 2025-11-04

**Authors:** Dionysios Tsoukalas, Lene Waldenstrøm, Jørgen Lerfall, John-Kristian Jameson, Philip James, Anita Nordeng Jakobsen

**Affiliations:** 1 Department of Biotechnology and Food Science, Norwegian University of Science and Technology (NTNU), Trondheim, Norway; 2 Food Microbiology, Wageningen University & Research (WUR), Wageningen, the Netherlands; 3 Nofima, Tromsø, Norway

**Keywords:** Agriculture, Fatty acids, Marine microbiology, Ocean sciences

## Abstract

The present study explored roe enhancement of the sea urchins *Echinus esculentus* (EC) and *Strongylocentrotus droebachiensis* (ST), over a 12-week trial using a formulated feed. While feeding did not significantly affect somatic growth or colorimetric parameters, it enhanced gonadal development, achieving a commercially acceptable gonad index and reducing hardness and chewiness. The feeding period improved nutritional profiles, increasing essential amino acids and beneficial fatty acids, while the sensory bitterness increased due to a rise in bitter-tasting free amino acids (FAA). A 2-week starvation period at the trial’s end reduced microbial counts by 1–2 log CFU/g. Inherent differences between species in colorimetric, textural and microbial parameters remained throughout the trial, with ST showing lighter, reddish, softer gonads. At the same time, EC had harder, brownish gonads, lower microbial counts, and higher chewiness. EC fed the formulated feed had increased gonads bitterness and stronger off-taste/aftertaste, likely due to higher bitter-tasting FAA levels.

## Introduction

Considered a luxury in the culinary world, sea urchin gonads, known as “uni” or “roe”, hold significant commercial value as a prized seafood delicacy^1^. According to the FAO^2^, global edible sea urchin capture has significantly declined from 109,736 tonnes (live weight) in 1995 to 54,626 tonnes (live weight) in 2022 due to overfishing and subsequent depletion of native stocks. Considering this decrease, efforts are being made to explore the feasibility of catch-based echinoculture to establish a more reliable source of sea urchin gonads. This method enhances gonad development by capturing wild sea urchins and feeding them artificial diets for several weeks prior to sale^3^.

While catch-based echinoculture traditionally focuses on marketable sea urchin species, exploring the roe enhancement optimization of underutilized species could offer a promising avenue for expanding the global sea urchin market. By diversifying sources and species, countries can mitigate pressure on overexploited stocks, while meeting consumer demands worldwide. *Echinus esculentus* and *Strongylocentrotus droebachiensis* are among the most abundant sea urchin species in the north and arctic areas, including Iceland, Norway, Ireland and Greenland^4^. *S. droebachiensis* is a commercially accepted species and frequently studied in roe enhancement trials due to its poor gonad development in the wild^5^. In contrast *E. esculentus* remains underutilized in the market despite its availability. Given the limited commercial exploitation of sea urchins in northern Europe, capturing wild individuals and feeding them for a few weeks for roe enhancement may present a promising strategy for developing a novel catch-based echinoculture business model.

Although *S. droebachiensis* and *E. esculentus* differ in biological and physiological traits, their overlapping geographic ranges enable a meaningful side-by-side comparison. The inclusion of *S. droebachiensis*, a species with established commercial relevance, provides a benchmark against which the potential of *E. esculentus* can be evaluated. Conducting roe enhancement on both species simultaneously under identical environmental conditions, a methodological approach rarely used in previous research, offers the opportunity to generate robust, directly comparable data on their responses to formulated diets. This controlled, comparative evaluation provides insights beyond those achievable in single-species studies and contributes valuable knowledge for the development of catch-based echinoculture practices.

To the best of our knowledge, research on commercial-sized *E. esculentus* remains limited, with Kelly et al.^6^ providing the only study on catch-based echinoculture that examined the effects of artificial diets on its biometric parameters. While *S. droebachiensis* has been relatively well studied in terms of how environmental factors (water quality, temperature, stocking density, and body weight) affect survival, gonad growth, feed intake, and feed conversion ratios in formulated diets^7^, far less attention has been given to the effects of artificial diet on gonad quality. The existing studies have examined only a narrow range of quality parameters^3,8–11^, indicating a clear need for more comprehensive research in this area. Thus, it is important to assess gonad quality as a multidimensional concept, considering various parameters such as size, taste, colour, texture, biochemical profile, microbial enumeration, and sensory assessment, to improve catch-based echinoculture practices and ensure the commercial viability of *E. esculentus* and *S. droebachiensis* in aquaculture.

Among the preferred characteristics of sea urchin gonads are a large size, vibrant colours ranging from bright red to yellowish and a moderate hardness^12^. Additionally, high-quality gonads boast a robust umami taste and a pleasant sweet flavour, which are closely attributed to their free amino acid concentrations^13^. Moreover, considering the increased consumer interest in high nutritional value and healthy products, the nutritional composition of gonads (proximate composition, fatty acid and total amino acid distribution) may serve as additional quality criteria. Also, Tsoukalas et al.^14^ highlighted the limited research on microbial analysis of gonads, emphasizing the importance of enumerating microbial counts in their quality assessment due to the common consumption of these products as raw or lightly processed.

The aim of the present study was to investigate the gonad quality during the roe enhancement of sea urchins *E. esculentus*, with *S. droebachiensis* serving as a commercially accepted reference species, by combining chemical, microbial and instrumental analyses along with a sensory assessment. Wild sea urchin individuals were harvested from Mid-Norway, maintained under identical tank conditions, and fed the same diet for 10 weeks, followed by 2 weeks of starvation. The physicochemical characteristics, biochemical composition, microbial counts and sensory attributes were investigated throughout the roe enhancement period and compared to those of pre-feeding wild-caught specimens, which serve as a biologically relevant baseline reflecting natural gonad status. This multidimensional and comparative assessment of gonad quality enhancement under controlled environmental and feeding regimes, could provide insights into the potential utilization of new sea urchin species in catch-based echinoculture, ultimately striving to produce gonads that meet consumer expectations.

## Results and discussion

This study investigated the physicochemical, biochemical, microbiological, and sensory attributes of the gonads of EC and ST sea urchins before and during a 12-week roe enhancement. The sea urchins were maintained in controlled, identical tank environments and fed a non-commercial algae-based feed previously used in roe enhancement experiments with *S. droebachiensis*, providing an established reference for evaluating *E. esculentus* performance. Tank effects were not significant for any measured parameter and are therefore not further considered. Moreover, a natural diet based solely on macroalgae was not included in the experiment, as previous studies have shown low gonad yield and quality in *S. droebachiensis*^15^ and other sea urchin species^16–18^ when fed fresh macroalgae. Beyond this, from a sustainability perspective, such a diet is less suitable for aquaculture^19^, as it requires additional harvesting rather than utilizing by-products.

The discussion follows a comparative framework built on three main pillars: evaluating the overall effect of feeding time on the examined parameters, exploring changes within each species over time and comparing them between species, and interpreting the findings in relation to pre-feeding wild-caught specimens as a biologically relevant baseline. This approach enables a comprehensive evaluation of species-specific responses to the formulated feed, offering insights rarely addressed in single-species studies. The study provides a holistic perspective on how roe enhancement with a formulated feed affects sea urchin quality, while also highlighting the potential of these species for catch-based echinoculture and their viability for large-scale commercial production.

### Survival rate

The survival rate was 93.9% for EC and 93% for SD, falling within the range previously reported for sea urchins fed on artificial diets^15,20^. Mortalities occurred sporadically over the roe enhancement period, with no noticeable peaks, particularly in the early days, indicating that initial transfer to the echinoculture system did not adversely affect survival through handling stress or other short-term factors (e.g., acclimation issues, dietary change). Water quality was unlikely to contribute to the observed mortalities as the ammonia levels remained under the detection limit and oxygen saturation was consistently above 90%^21^. The similar survival rates between species further suggest no species-specific differences in resilience under the experimental conditions. Instead, the mortalities are likely linked to behavioural interactions. The sea urchins spent most of their time attached to the vertical wall surfaces of the tanks and descended only briefly to the bottom to access feed. During these movements, individuals collided with one another, leading to injuries that, in some cases, proved fatal. This interpretation aligns with observations that sea urchins returned to vertical surfaces after feeding, suggesting that mortality was associated with these short but frequent vertical movements rather than environmental stress or poor water quality^21^.

### Biometric parameters and production efficiency

**Fig. 1:**
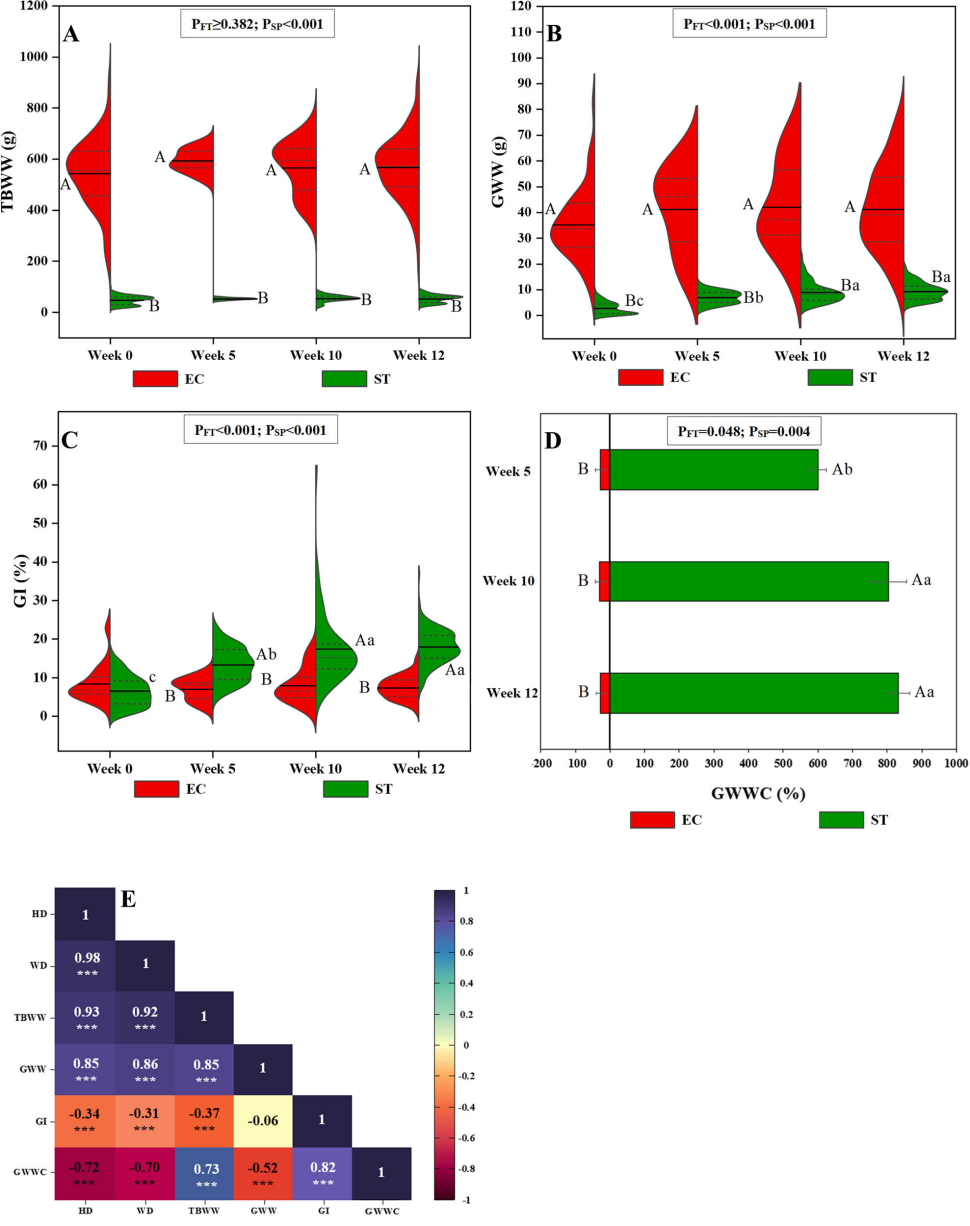
Biometric parameters. Split violin boxplots of total body wet weight (TBWW) (g) (**A**), gonad wet weight (GWW) (g) (**B**), gonad index (GI) (%) (**C**), pyramid plot of gonad wet weight change (GWWC) (%) compared to week 0 (**D**) and correlation heatmap between biometrical parameters (**E**) of *Echinus esculentus* (EC) and *Strongylocentrotus droebachiensis* (ST) gonads. In the split violin boxplots, the narrow dashed lines visualize 25–75% of specimens values, wide dash lines (median), and solid lines (mean). In the pyramid plot, the error bars represent one standard deviation. In the correlation heatmap, the HD Height diameter, WD Width diameter; ***P < 0.001. *P*_FT_ and *P*_SP_ are the significant levels (GLM) for the effects of feeding time and species, respectively. ^a–c^Different lowercase letters within each species indicate significant differences (*P* < 0.05) in biometric parameters throughout feeding time. ^A-B^Different uppercase letters within each feeding time indicate significant differences (*P* < 0.05) in biometric parameters between the species at the same feeding time. This graph was created with OriginPro 2022 (OriginLab Corp.).

Biometrical parameters are essential indicators of sea urchin quality, directly affecting the quality attributes and overall potential for commercial use. No significant differences were found on WD (*P*_FT_ ≥ 0.529), HD (*P*_FT_ ≥ 0.780) and TBWW (*P*_FT_ ≥ 0.382) within each sea urchin species (EC and ST) throughout the roe enhancement (Fig. 1, Supplementary Table S1). The measured values remained similar to those recorded at the pre-feeding baseline specimens (week 0), aligning with previous studies on comparably sized sea urchin species fed for 8–12 weeks^22,23^, and suggesting that the relatively short feeding duration was insufficient to promote substantial somatic growth.

Nevertheless, feeding time significantly affected the GWW (*P*_FT_ < 0.001), GWWC (*P*_FT_ = 0.048), and the GI (*P*_FT_ < 0.001) in both species (Fig. 1, Supplementary Table S1). The observed increase in GWW indicates significant gonadal growth compared to pre-feeding baseline specimens, aligning with previous research on various sea urchins, which has consistently reported increased gonadal development in response to feeding with artificial diets^11,15,17,24^. However, the higher the GWW, the smaller the increase in GWWC as demonstrated by the inverse correlation between GWW and GWWC (*r* = −0.52, *P* < 0.001) (Fig. 1E).

Moreover, significant differences were found between the species in all the biometric parameters (*P*_SP_ < 0.001) (Fig. 1, Supplementary Table S1). The average GWW of ST significantly increased from 2.9 ± 2.3 g at the pre-feeding baseline specimens to 9.0 ± 4.1 g in week 10 of roe enhancement, after which it remained constant during the two-week starvation (Fig. 1B). In comparison, the average GWW of EC increased from 35.2 ± 13.7 g to 41.2 ± 16.2 g by the 5^th^ week and then remained stable. However, this increase was not statistically significant. As a result, by the end of roe enhancement, the GWWC of ST had significantly increased by ~830%, while EC showed a smaller, non-significant GWWC of 18% (Fig. 1D). In addition, an inverse correlation was observed between HD, WD and TBWW with GWWC (*r* = −0.70 to −0.73, *P* < 0.001) (Fig. 1E). This difference in GWWC between the species aligns with findings in other sea urchin species, where gonadal production was inversely correlated with body size, with smaller individuals showing the greatest percentage increases in GWWC^15,18^. The allometric relationship may stem from larger individuals dedicating more energy to maintenance rather than gonad development, consuming less food, exhibiting lower absorption or assimilation efficiency, and requiring more energy to achieve the same proportional increase in gonad yield^15^.

Beyond body size, environmental and biological factors may also contribute to interspecific differences in GWWC. Seasonality is one such factor, as gonad development in wild populations is strongly influenced by seasonal cycles^25^; however, when roe enhancement is conducted under constant, stable temperatures, the season of harvest appears to have minimal effect on subsequent growth^26^. In addition, pre-capture nutritional condition may also affect GWWC, since feed availability in the natural habitat can influence baseline gonad size^25^. In the present study, this factor is unlikely here since both species were collected from the same location, although potential interspecific differences in feeding behaviour or foraging efficiency prior to capture cannot be entirely ruled out and warrant further investigation. Moreover, the reproductive cycle is another consideration: in Norway, ST typically spawns in April/May^27^, while EC likely spawns later in summer, around August^28^. This difference in timing could explain the greater GWWC increase in ST, which may have been in a spawning or post-spawning phase, as indicated by low pre-feeding GWW, enabling greater proportional gains, whereas EC may have been closer to maturity. Determining gonad maturity stages was beyond the scope of this study but should be addressed in future work.

Regarding the GI, ST exhibited a significant gradual increase throughout the roe enhancement period, rising from 6.5 ± 4% at pre-feeding baseline specimens to 17.7 ± 6.7% at the end of feeding trial. Its final GI surpassed the generally recognized marketable size of 15%, making it commercially acceptable and aligning with findings from comparable artificial diet experiments conducted with either ST^11,15^ or other sea urchin species^22,29^. In contrast, the GI of EC did not show a significant increase, remaining an average of 7.3 ± 3.6% throughout the trial. This implies that a small increase in the GWW of larger size sea urchins, like EC in our study, may not always be reflected in the GI. Moreover, an inverse correlation was observed between HD, WD and TBWW with GI (*r* = −0.31 to −0.37, *P* < 0.001) (Fig. 1E). Similar inverse correlations between GI and size have been observed in wild EC^28^ and ST fed formulated feed^15^. Nonetheless, EC’s relatively high initial and final GWW still positions it as a profitable size for the market, surpassing the GWW of ST, a species already established in commercial markets.

Building on these measurements, GPE was assessed as a performance indicator, offering a more accurate reflection of feed conversion toward gonad biomass than metrics such as specific growth rate or feed conversion ratio, which can be confounded by the high proportion of coelomic fluid in sea urchins^30^. GPE varied significantly throughout the roe enhancement period (*P*_FT_ < 0.001) (Supplementary Table S1), with values comparable to those reported in similar studies using artificial diets^31–33^. During the first five weeks of feeding, GPE reached its highest values, reflecting a phase of rapid gonad biomass accumulation. This early surge likely results from the transition of pre-feeding specimens from a natural environment with limited food availability to conditions of abundant feed, promoting reproductive growth before energy allocation gradually shifts toward somatic maintenance. The pattern is reinforced by GWWC data, which also peaked during the initial feeding period. The strong positive correlation between GWWC and GPE (*r* = 0.60, *P* < 0.001) supports the conclusion that early feeding represents a period of maximal gonadal deposition efficiency. These findings are further supported by literature indicating that adequate nutrient availability promotes resource allocation toward gonadal development rather than somatic maintenance^33^.

Moreover, significant differences in the GPE was observed between the species (*P*_SP_ = 0.033) with EC have significant lower GPE than ST in both time intervals. The lower GPE in EC was not unexpected, as this species entered the trial with already well-developed gonads, leaving less capacity for further substantial increases. In addition, the larger overall body size of EC likely increased its baseline energy requirements for maintenance, reducing the proportion of dietary energy available for gonad growth^15^.

The results of this study demonstrate that, despite the absence of significant somatic growth, roe enhancement effectively promoted gonadal development in both species compared to the pre-feeding baseline. Production efficiency analysis further confirmed that a substantial proportion of ingested feed was converted into gonad biomass, particularly during the early stages of feeding. While inherent species-specific differences in biometric traits remain, the roe enhancement resulted in commercially acceptable GI and GWW for both species, indicating that EC has a potential comparable to the commercially established ST for catch-based echinoculture.

### Colorimetric parameters

**Fig. 2:**
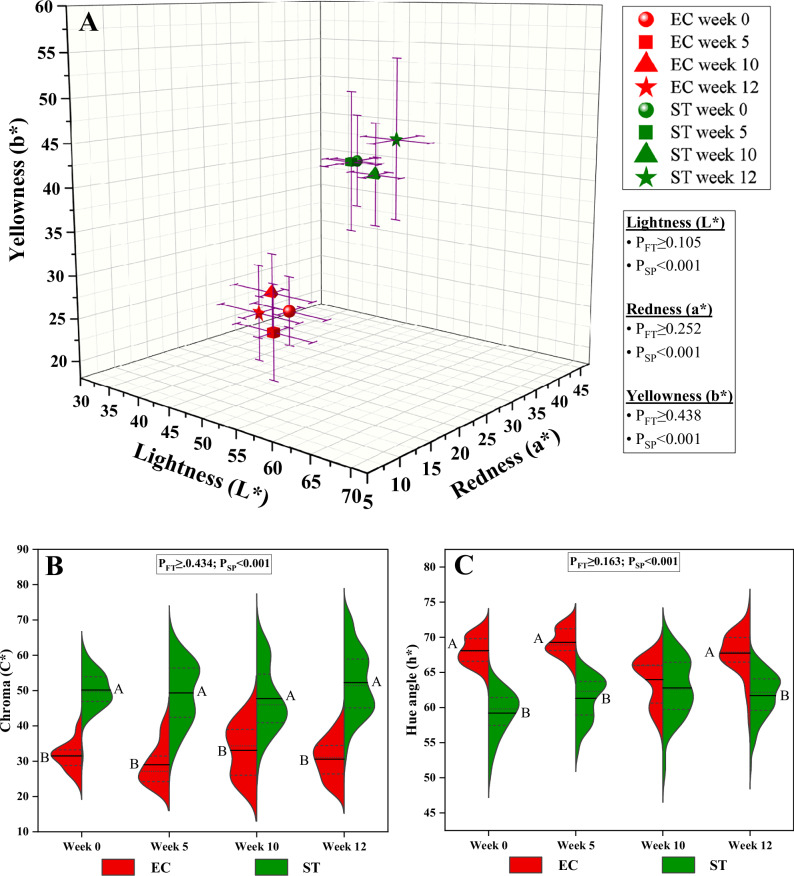
Colorimetric parameters. 3D scatter plot illustrating the lightness (L*), redness (a*) and yellowness (b*) (**A**) and split violin boxplots of Chroma (C*) (**B**) and hue angle (h*) (**C**) of *Echinus esculentus* (EC) and *Strongylocentrotus droebachiensis* (ST) gonads. In the 3D plot, the error bars represent one standard deviation. In the split violin boxplots, the narrow dashed lines visualize 25–75% of specimens values, wide dash lines (median), and solid lines (mean). *P*_FT_ and *P*_SP_ are the significant levels (GLM) for the effects of feeding time and species, respectively. ^A-B^Different uppercase letters within each feeding time indicate significant differences (*P* < 0.05) in C* and h* between the species at the same feeding time. This graph was created with OriginPro 2022 (OriginLab Corp.).

Gonad colour is a crucial factor in determining the market appeal and quality of sea urchins, primarily derived from carotenoid pigments like echinenone, synthesized from β-carotene in the diet^15^. In the present study, no significant differences in colorimetric parameters were observed (*P*_FT_ ≥ 0.105) within each sea urchin species (EC and ST) throughout the roe enhancement period compared to the pre-feeding baseline specimens (Fig. 2, Supplementary Table S2), indicating limited impact of the formulated feed on gonad coloration. These findings align with a similar study on ST fed artificial diets for 60 days^3^.

In addition, significant differences were observed between the species in all examined parameters (*P*_SP_ < 0.001) (Fig. 2, Supplementary Table S2). In the pre-feeding baseline specimens (week 0), the gonads of EC were more translucent (L* = 56.3 ± 5.1), less reddish (a* = 11.7 ± 1.9) and less yellowish (b* = 29.2 ± 3.7) than those of ST (L* = 53.9 ± 3.4; a* = 25.6 ± 2.9; b* = 43.0 ± 5.1) (Fig. 2A). Moreover, EC displayed lower Chroma value (C* = 31.5 ± 3.9) and higher hue angles (h* = 68.1 ± 2.0), indicating a paler and more brownish colour than ST (C* = 50.1 ± 5.3; h* = 59.2 ± 3.1) (Fig. 2B, C, Supplementary Table S2). In addition, the colorimetric attributes varied not only between species but also within species (as shown by the relatively high standard deviation), due to inherent differences and individual variability. These differences suggest that EC and ST naturally exhibit distinct gonad coloration patterns, which could influence market appeal and may reflect species-specific pigmentation and physiological traits.

At the end of the trial, ST were lighter than EC (L* = 60.1 ± 4.5 and 52.1 ± 5.8, respectively), with lightness showing an inverse correlation with sea urchin size (WD and HD) (*r* = −0.44, *P* < 0.001), consistent with the inverse relationship reported in previous studies^15,22^. Nevertheless, colour parameters for both EC and ST, before and after roe enhancement, fell within the range reported in other studies^15,17,22,34,35^. Since market preferences for gonad colour can vary, with acceptable hues ranging from bright yellow to dark orange or red, with pale or dark brown gonads being generally less desirable^36^, the artificial diet and the relatively short feeding period was not able to enhance EC gonad coloration to reach levels comparable with the well-established commercially acceptable ST gonads, which still remains more appealing for the market.

### Texture parameters

Maintaining the integrity of gonads is vital for ensuring high-quality gonad products, with hardness standing out as a paramount attribute among quality parameters. Among the texture parameters, the hardness and chewiness were significantly affected by the feeding time (*P*_FT_ < 0.001 and *P*_FT_ = 0.013, respectively), showing a marked reduction (30–40%) by week 5 in both species compared to initial values of the pre-feeding baseline specimens. No further significant changes were observed for the remainder of the roe enhancement period (Table 1). This decline in hardness is consistent with previous findings^17^, where ST gonads exhibited better texture prior to artificial diet feeding. Additionally, the inverse correlations detected between hardness and both GI and GWWC (*r* = −0.39 and −0.79, respectively; *P* < 0.001), suggesting that the reduction in hardness may be associated with increased GWWC and GI during feeding. These results support previous studies indicating that gonadal growth^17,37^ and rising GI^35^ are generally linked to softer gonads.

**Table 1:** Texture profile analysis parameters (mean ± SD) of *Echinus esculentus* (EC) and *Strongylocentrotus droebachiensis* (ST) gonads

Species	Feeding time	Hardness (N)	Cohesiveness	Springiness (mm)	Chewiness (mJ)
EC	Week 0	1.28 ± 0.11^Aa^	0.36 ± 0.06	0.83 ± 0.08	0.38 ± 0.09^Aa^
	Week 5	0.88 ± 0.10^Ab^	0.31 ± 0.04	0.82 ± 0.08	0.22 ± 0.05^Ab^
	Week 10	0.87 ± 0.09^Ab^	0.31 ± 0.03	0.80 ± 0.07	0.22 ± 0.04^Ab^
	Week 12	0.86 ± 0.11^Ab^	0.30 ± 0.11	0.77 ± 0.11	0.20 ± 0.09^Ab^
ST	Week 0	0.84 ± 0.06^Ba^	0.33 ± 0.04	0.78 ± 0.04	0.22 ± 0.04^Ba^
	Week 5	0.54 ± 0.05^Bb^	0.31 ± 0.06	0.76 ± 0.09	0.13 ± 0.03^Bb^
	Week 10	0.55 ± 0.07^Bb^	0.30 ± 0.07	0.73 ± 0.07	0.12 ± 0.04^Bb^
	Week 12	0.54 ± 0.07^Bb^	0.30 ± 0.05	0.75 ± 0.11	0.12 ± 0.04^Bb^
GLM^*^	P_FT_	<0.001	0.261	0.208	0.013
	P_SP_	<0.001	0.365	0.293	<0.001

*P*_FT_ and *P*_SP_ are the significant levels (GLM) for the effects of feeding time and species, respectively.

^*^General linear model (GLM) analysis of variance with feeding time and species as fixed factors; where significant differences were detected (*P* < 0.05), a Tukey’s pairwise comparison or a *t*-test were applied.

^a–b^Different superscript lowercase letters within each species and each parameter indicate significant differences (*P* < 0.05) throughout feeding time.

^A-B^Different superscript uppercase letters within each feeding time and each parameter indicate significant differences (*P* < 0.05) between the species at the same feeding time.

Regarding the sea urchin species, EC gonads exhibited significantly higher hardness and chewiness than those of ST (*P*_SP_ < 0.001) (Table 1), both at the pre-feeding baseline specimens and throughout the roe enhancement, with GWW positively correlated with both hardness (*r* = 0.48, *P* < 0.001) and chewiness (*r* = 0.25, *P* < 0.001). The discrepancy between the species may be attributed to the substantial difference in GWW (35–41 g for EC vs. 2–9 g for ST), suggesting that larger, well-developed gonads of EC contributed to their firmer texture. However, factors such as intracellular spacing, membrane fluidity, and reproductive cycle may also play a role^15^. Additionally, the strong correlation between hardness and chewiness (*r* = 0.84, *P* < 0.001) likely accounts for the observed species-specific differences.

In general, the observed decrease in the textural parameters relative to the pre-feeding baseline specimens could be considered a negative aspect for commercial roe industry, with the softer texture in ST presenting an even greater challenge. In comparison, EC gonads displayed firmer and chewier attributes, suggesting superior texture quality relative to the commercially well-established ST gonads. Soft roe can be firmed by soaking the gonads in chilled brine containing aluminium sulphate^10,38^. However, the use is restricted in the EU^39^, and alternative methods should be investigated. In cases where roe texture quality is unsuitable, alternative applications such as marinated or canned products, or using sea urchin as a spice or ingredient in culinary formulations, can be considered.

### Gonad pH

Apart from initial pH values reported in some sea urchin feeding studies, consistent measurement of gonad pH during feeding trials appears to be absent from the literature. In this study, gonad pH was included as a less common physiological parameter to provide additional insights into the biochemical changes occurring in the gonads in response to diet. The initial gonad pH values of both species (6.3–6.4) at the pre-feeding baseline specimens align with those reported for various sea urchin species^40,41^, with no significant differences between the species (*P*_SP_ ≥ 0.905) during the roe enhancement. However, gonad pH gradually increased throughout the roe enhancement trial (*P*_FT_ < 0.001) from an average of 6.4 ± 0.2 to 6.8 ± 0.1 (Table 2). This shift may be attributed to the potential greater assimilation efficiency of protein in the artificial diet, which likely led to elevated ammonia production and subsequent increase in gonad pH.

**Table 2:** Proximate composition and pH (mean ± SD) of *Echinus esculentus* (EC) and *Strongylocentrotus droebachiensis* (ST) gonads

Species	Feeding time	Water (%)	Ash (%)	Proteins (%)	Lipids (%)	pH
EC	Week 0	82.8 ± 1.6^a^	2.3 ± 0.3^a^	11.0 ± 0.5	2.6 ± 0.4^c^	6.4 ± 0.1^c^
	Week 5	82.8 ± 2.3^Aa^	2.3 ± 0.3^Aa^	11.1 ± 0.9	3.1 ± 0.4^Bb^	6.6 ± 0.2^bc^
	Week 10	82.8 ± 1.4^Aa^	1.9 ± 0.2^b^	10.8 ± 0.7	3.2 ± 0.2^Bab^	6.7 ± 0.2^ab^
	Week 12	81.3 ± 0.8^Ab^	1.8 ± 0.1^b^	10.7 ± 0.4	3.7 ± 0.4^a^	6.8 ± 0.1^a^
ST	Week 0	81.1 ± 2.4^a^	2.3 ± 0.2^a^	11.2 ± 1.3	2.7 ± 0.2^c^	6.3 ± 0.2^c^
	Week 5	80.8 ± 1.5^Ba^	1.9 ± 0.3^Bb^	11.1 ± 0.7	3.4 ± 0.4^Ab^	6.6 ± 0.2^b^
	Week 10	80.8 ± 1.8^Ba^	1.9 ± 0.6^b^	11.3 ± 0.3	3.8 ± 0.1^Aab^	6.6 ± 0.1^b^
	Week 12	79.7 ± 2.2^Bb^	1.7 ± 0.6^b^	10.8 ± 0.6	4.0 ± 0.4^a^	6.9 ± 0.1^a^
GLM^*^	P_FT_	0.014	<0.001	0.983	<0.001	<0.001
	P_SP_	<0.001	0.028	0.301	0.002	0.905

*P*_FT_ and *P*_SP_ are the significant levels (GLM) for the effects of feeding time and species, respectively.

^*^General linear model (GLM) analysis of variance with feeding time and species as fixed factors; where significant differences were detected (*P* < 0.05), a Tukey’s pairwise comparison or a t-test were applied.

^a–c^Different superscript lowercase letters within each species and each parameter indicate significant differences (*P* < 0.05) throughout feeding time.

^A-B^Different superscript uppercase letters within each feeding time and each parameter indicate significant differences (*P* < 0.05) between the species at the same feeding time.

### Proximate composition

The proximate composition, including fatty acid distribution and amino acid composition, has a direct influence on the nutritional value and sensory quality of sea urchin gonads. The constant protein content in the gonads of both species (averaging 11.1 ± 0.7%) during the roe enhancement (*P*_FT_ ≥ 0.983) (Table 2) is in line with previous research on ST^32^, *Paracentrotus lividus*^42^, and *Mesocentrotus franciscanus*^43^, which reported that gonadal protein levels remain stable regardless of dietary protein intake.

However, the water, ash and lipid content were significantly affected by roe enhancement (*P*_FT_ = 0.014, *P*_FT_ < 0.001 and *P*_FT_ < 0.001, respectively) (Table 2). During the feeding period, the ash content significantly decreased from an average of 2.3 ± 0.2% at the pre-feeding baseline specimens to 1.8 ± 0.2% at the end of roe enhancement, while the average lipid content significantly increased from 2.7 ± 0.3% to 3.9 ± 0.4%. Although water content slightly decreased over the roe enhancement, a significant reduction was only observed in the last two weeks of starvation. Moreover, a correlation was noted between lipid content and both ash content (*r* = −0.44, *P* < 0.001) and water content (*r* = −0.27, *P* = 0.007). Since the formulated feed had a relatively low lipid concentration (1.7 ± 0.1%), the increase in gonadal lipid content during artificial diet feeding was most likely due to the significant reduction in water and ash content.

When comparing the species, significant differences were found between them in the water, ash and lipid content (*P*_SP_ < 0.001, *P*_SP_ = 0.028 and *P*_SP_ = 0.002, respectively) during the roe enhancement. However, the proximate composition was comparable between the pre-feeding baseline specimens and those at the end of the feeding period (except the water content after feeding), indicating that the diet had no effect on the fundamental components of either species gonads. Consequently, both species could be considered as a good source of protein (10.7–11.1%) with moderate lipid content (2.6–4.0%), highlighting that EC matches the commercially accepted ST in these fundamental attributes.

### Fatty acids distribution

The concentration of most fatty acids was significantly influenced by roe enhancement (Table 3). In both sea urchin species fed the formulated feed, a shift in their fatty acid profile was observed during the roe enhancement, characterized by a reduction in total saturated fatty acids (ΣSFA; from 29.8 ± 1.4% to 25.8 ± 1.3%, *P*_FT_ < 0.001) and a concurrent increase in total monounsaturated (ΣMUFA; from 20.6 ± 0.9% to 23.7 ± 0.7%, *P*_FT_ < 0.001) and total polyunsaturated fatty acids (ΣPUFA; from 44.4 ± 1.4% to 49.3 ± 0.7%, *P*_FT_ < 0.001) compared to pre-feeding baseline specimens. Moreover, these alterations in ΣSFA, ΣMUFA, and ΣPUFA were evenly distributed across various fatty acids. A similar pattern has been observed in other studies on fed sea urchins, including ST^8,44,45^. However, the fluctuations in individual fatty acids distribution during roe enhancement are not entirely comparable between studies, with variations likely arising from a range of endogenous and exogenous factors such as inherent differences in the initial fatty acid composition of wild specimens gonads, the type of natural versus artificial diets, and the extent of de novo fatty acid synthesis.

**Table 3:** Fatty acid (%) distribution (mean ± SD) of *Echinus esculentus* (EC) and *Strongylocentrotus droebachiensis* (ST) gonads

	EC				SP				GLM^a^	
	Week 0	Week 5	Week 10	Week 12	Week 0	Week 5	Week 10	Week 12	*P*_FT_	P_SP_
*Saturated fatty acids*										
C14:0	4.0 ± 0.4^Ba^	3.7 ± 0.4^Bab^	3.5 ± 0.4^Bab^	3.4 ± 0.3^Bb^	12.5 ± 1.2 ^Aa^	10.6 ± 1.3^Ab^	10.4 ± 0.3^Ab^	10.1 ± 0.5^Ab^	<0.001	<0.001
C15:0	1.2 ± 0.1^A^	1.0 ± 0.4	0.9 ± 0.3	0.8 ± 0.1	0.6 ± 0.1^B^	0.9 ± 0.3	0.9 ± 0.1	0.9 ± 0.1	0.544	0.005
C16:0	12.1 ± 1.2^A^	12.1 ± 0.7 ^A^	11.6 ± 0.4 ^A^	11.3 ± 0.3 ^A^	5.5 ± 0.5^Ba^	4.2 ± 0.2^Bb^	4.2 ± 0.2^Bb^	4.3 ± 0.3^Bb^	<0.001	<0.001
C18:0	3.7 ± 0.2 ^A^	3.2 ± 0.4 ^A^	3.3 ± 0.2 ^A^	3.4 ± 0.3 ^A^	2.2 ± 0.2^B^	2.7 ± 0.3^B^	2.2 ± 0.2^B^	2.2 ± 0.1^B^	0.109	<0.001
C20:0	8.4 ± 0.3^Ba^	8.6 ± 0.4^Ba^	7.2 ± 0.7^Bb^	7.2 ± 0.4^b^	9.4 ± 0.5 ^Aa^	9.3 ± 0.3 ^Aa^	8.6 ± 0.4 ^Aab^	7.9 ± 1.5^b^	<0.001	<0.001
ΣSFA	29.4 ± 1.0^a^	28.5 ± 1.0^a^	26.5 ± 0.9^b^	26.2 ± 0.5^b^	30.2 ± 1.7^a^	27.7 ± 1.1^b^	26.4 ± 0.5^bc^	25.4 ± 1.8^c^	<0.001	0.437
*Monounsaturated fatty acids*										
C14:1	1.1 ± 0.2^b^	1.5 ± 0.1^Aa^	1.6 ± 0.1^Aa^	1.4 ± 0.1^Aa^	1.2 ± 0.2	1.0 ± 0.3^B^	1.0 ± 0.1^B^	1.0 ± 0.2^B^	0.224	<0.001
C16:1 n-7	4.9 ± 0.5^A^	5.0 ± 0.3^A^	4.8 ± 0.4 ^A^	4.8 ± 0.4^A^	3.4 ± 0.5^B^	3.0 ± 0.4^B^	3.0 ± 0.2^B^	3.0 ± 0.1^B^	0.123	<0.001
C17:1	0.3 ± 0.1 ^A^	0.8 ± 0.2	0.8 ± 0.1 ^A^	0.8 ± 0.1	0.2 ± 0.1^Bb^	0.7 ± 0.1^a^	0.7 ± 0.1^Ba^	0.7 ± 0.1^a^	<0.001	0.004
C18:1 n-9	3.7 ± 0.3^Bc^	3.5 ± 0.4^Bc^	5.4 ± 0.5 ^Ab^	6.8 ± 0.9^Aa^	4.2 ± 0.5^A^	4.4 ± 0.7 ^A^	4.3 ± 0.2^B^	4.4 ± 0.2^B^	<0.001	<0.001
C20:1	6.9 ± 0.3^Ba^	5.9 ± 0.5^Bb^	7.0 ± 0.5^Ba^	6.5 ± 0.4^Bab^	9.1 ± 0.6^Ab^	11.9 ± 0.6^Aa^	12.1 ± 0.4 ^Aa^	12.4 ± 0.5^Aa^	<0.001	<0.001
C22:1	3.5 ± 0.4^A^	3.3 ± 0.3 ^A^	3.7 ± 0.3 ^A^	3.5 ± 0.3 ^A^	2.8 ± 0.5^Ba^	2.2 ± 0.5^Bb^	2.2 ± 0.1^Bb^	2.1 ± 0.1^Bb^	0.009	<0.001
ΣMUFA	20.3 ± 0.6^b^	20.0 ± 0.8^Bb^	23.2 ± 0.9^a^	23.7 ± 0.8^a^	20.9 ± 1.1^b^	23.2 ± 1.2^Aa^	23.2 ± 0.6^a^	23.6 ± 0.6^a^	<0.001	<0.001
*Polyunsaturated fatty acids*										
C16:2 n-4	2.8 ± 0.6^a^	2.1 ± 0.3^Bb^	3.1 ± 0.3^Ba^	3.2 ± 0.3^Ba^	3.2 ± 0.5^b^	3.7 ± 0.4 ^Aab^	4.0 ± 0.2^Aa^	4.2 ± 0.5^Aa^	<0.001	<0.001
C18:4 n-3	0.1 ± 0.1^Bb^	1.3 ± 0.1^Bab^	2.0 ± 0.1^a^	2.0 ± 0.1^Ba^	1.5 ± 0.5^Ab^	1.9 ± 0.2^Aa^	2.0 ± 0.1^a^	2.2 ± 0.2^Aa^	<0.001	<0.001
C20:2 n-6	3.8 ± 0.3^b^	4.4 ± 0.4^a^	4.4 ± 0.3^a^	4.4 ± 0.2^a^	4.2 ± 0.4	4.3 ± 0.4	4.2 ± 0.3	4.0 ± 0.4	0.018	0.612
C20:3	10.7 ± 0.6^b^	11.3 ± 1.0^Bab^	11.5 ± 0.5^Bab^	11.7 ± 0.5^Ba^	11.5 ± 1.0^b^	12.3 ± 0.6^Aab^	12.9 ± 0.5^Aa^	12.6 ± 0.4 ^Aa^	<0.001	<0.001
C20:4	1.8 ± 0.2	2.0 ± 1.0	2.2 ± 0.1	2.2 ± 0.2	2.3 ± 0.1	2.1 ± 0.3	2.2 ± 0.1	2.2 ± 0.2	0.434	0.164
C20:5 n-3 (EPA)	16.0 ± 0.7^b^	16.1 ± 0.9^Bb^	17.3 ± 0.6^a^	17.6 ± 0.6^a^	16.6 ± 0.5^b^	17.7 ± 1.1^Aa^	17.7 ± 0.3^a^	18.1 ± 0.6^a^	<0.001	<0.001
C22:6 n-3 (DHA)	8.4 ± 0.4^A^	8.4 ± 0.4^A^	8.4 ± 0.3 ^A^	8.3 ± 0.4 ^A^	5.9 ± 0.3^B^	5.5 ± 0.4^B^	5.8 ± 0.3^B^	5.8 ± 0.4^B^	0.340	<0.001
ΣPUFA	43.7 ± 0.9^Bc^	45.6 ± 1.4^Bb^	48.8 ± 0.4^a^	49.3 ± 0.7^a^	45.2 ± 1.4^Ab^	47.6 ± 2.0^Aa^	48.8 ± 0.8^a^	49.2 ± 0.8^a^	<0.001	0.007

*P*_FT_ and *P*_SP_ are the significant levels (GLM) for the effects of feeding time and species, respectively.

*General linear model (GLM) analysis of variance with feeding time and species as fixed factors; where significant differences were detected (*P* < 0.05), a Tukey’s pairwise comparison or a t-test were applied.

^a–c^Different superscript lowercase letters within each species and each fatty acid indicate significant differences (*P* < 0.05) throughout feeding time.

^A-B^Different superscript uppercase letters within each feeding time and each fatty acid indicate significant differences (*P* < 0.05) between the species at the same feeding time.

Although no differences were observed in ΣSFA between the species (*P*_SP_ ≥ 0.437), EC contained notably higher levels of C16:0 and lower levels of C14:0 compared to ST, both at the pre-feeding baseline specimens and throughout the roe enhancement. Similarly, ΣMUFA levels were comparable between species at the pre-feeding baseline (*P*_SP_ ≥ 0.204) and at the end (*P*_SP_ ≥ 0.261) of roe enhancement, but EC had a considerably higher proportion of C16:1 n-7 and lower C20:1 than ST. While EC had significantly higher ΣPUFA levels until week 5 (*P*_SP_ ≤ 0.039), both species displayed similar ΣPUFA concentrations thereafter (*P*_SP_ ≥ 0.376), primarily composed of C20:3, eicosapentaenoic acid (EPA), and docosahexaenoic acid (DHA), with EC showing a notably higher accumulation of DHA. The characteristic fatty acid profile observed in both species aligns with those documented in the literature, with similar dominant fatty acids within the SFA, MUFA, and PUFA categories and comparable relative abundances^8,28,40,45^. As such, these findings emphasize the potential of both EC and the commercially well-established ST, whether fed formulated feed or not, as valuable sources of beneficial MUFAs and PUFAs. However, it should be noted that including the fatty acid composition of the feed could have provided additional insights, although disclosure is restricted due to confidentiality agreements.

### Total amino acids and free amino acids distribution

As observed for fatty acids, the distribution of most TAA was significantly affected by the roe enhancement (Table 4). In both species, a significant rise in the total essential amino acids (ΣEAA) alongside a corresponding notable decrease in the total non-essential amino acids (ΣNEAA) was observed (*P*_FT_ < 0.001) relative to the pre-feeding baseline specimens, indicating a positive impact of the formulated feed on the gonads’ nutritional profile. This shift is further supported by the steady increase in the total essential amino acids/total non-essential amino acids (ΣEAA/ΣNEAA) ratio (from an average of 0.46 ± 0.03 to 0.61 ± 0.09, *P*_FT_ < 0.001) throughout the feeding trial, approaching 60%, which is considered a benchmark for high-quality protein^46^.

**Table 4:** Total animo acids (TAA) (%) distribution (mean ± SD) of *Echinus esculentus* (EC) and *Strongylocentrotus droebachiensis* (ST) gonads

	EC				SP				GLM**	
Amino acids	Week 0	Week 5	Week 10	Week 12	Week 0	Week 5	Week 10	Week 12	*P*_FT_	*P*_SP_
*Essential*										
Histidine	1.3 ± 0.1^b^	1.7 ± 0.3^Aa^	1.5 ± 0.2^ab^	1.6 ± 0.2^ab^	1.4 ± 0.3^b^	1.4 ± 0.2^Bb^	1.5 ± 0.2^ab^	1.8 ± 0.2^a^	0.003	0.905
Isoleucine	3.8 ± 0.1^Ac^	3.6 ± 0.3^c^	4.7 ± 0.5^Ab^	5.3 ± 0.3^Aa^	3.3 ± 0.3^Bc^	3.8 ± 0.4^b^	3.7 ± 0.2^Bb^	4.3 ± 0.2^Ba^	<0.001	<0.001
Leucine	6.2 ± 0.4^Ac^	6.4 ± 0.5^c^	8.2 ± 0.5^Ab^	9.0 ± 0.7^Aa^	5.7 ± 0.4^Bc^	6.3 ± 0.3^b^	6.2 ± 0.4^Bbc^	6.9 ± 0.3^Ba^	<0.001	<0.001
Lysine	6.5 ± 0.5	7.0 ± 0.9	6.3 ± 0.7^B^	6.4 ± 0.3	6.7 ± 0.7	6.5 ± 0.3	7.1 ± 0.6 ^A^	6.5 ± 0.3	0.413	0.402
Methionine	1.8 ± 0.2^c^	2.1 ± 0.2^bc^	2.3 ± 0.4 ^Aab^	2.6 ± 0.4^Aa^	1.8 ± 0.2^b^	2.2 ± 0.2^a^	1.8 ± 0.3^Bb^	2.1 ± 0.3^Bab^	<0.001	0.002
Phenylalanine	3.3 ± 0.2^b^	3.5 ± 0.2^Bb^	4.9 ± 0.5^Aa^	4.9 ± 0.5^Aa^	3.3 ± 0.2^b^	3.8 ± 0.3 ^Aa^	3.6 ± 0.3^Bab^	3.9 ± 0.3^Ba^	<0.001	<0.001
Threonine	4.1 ± 0.4^B^	4.0 ± 0.3^B^	4.4 ± 0.3	4.3 ± 0.1	4.6 ± 0.4^A^	4.4 ± 0.3^A^	4.5 ± 0.3	4.3 ± 0.3	0.341	0.001
Valine	5.1 ± 0.3^Abc^	4.7 ± 0.4^c^	5.7 ± 0.6^Ab^	6.9 ± 0.6^Aa^	3.9 ± 0.4^Bc^	4.4 ± 0.3^b^	4.6 ± 0.2^Bb^	5.0 ± 0.3^Ba^	<0.001	<0.001
ΣEEA	32.0 ± 1.3^c^	33.1 ± 1.6^c^	37.9 ± 2.4^Ab^	40.9 ± 2.3^Aa^	30.8 ± 1.5^c^	32.8 ± 1.1^b^	32.9 ± 1.5^Bb^	34.8 ± 0.5^Ba^	<0.001	<0.001
*Non-essential*										
Asparagine	6.6 ± 0.2^Ba^	6.7 ± 0.5^a^	6.2 ± 0.6^Bab^	5.8 ± 0.7^Bb^	7.0 ± 0.5^Aab^	7.1 ± 0.6^a^	7.0 ± 0.4^Aab^	6.5 ± 0.3^Ab^	<0.001	<0.001
Glutamine	<0.05^a^	<0.04^Aab^	<0.02^b^	<0.03^ab^	<0.02^ab^	<0.01^Bb^	<0.01^b^	<0.04^a^	0.007	<0.001
Arg/Glyc^a^	33.9 ± 1.4^a^	32.3 ± 1.9^Aa^	23.5 ± 5.1^Bb^	25.1 ± 2.8^Bb^	32.4 ± 2.0^a^	29.4 ± 0.9^Bb^	29.5 ± 0.9^Ab^	28.6 ± 1.3^Ab^	<0.001	0.040
Tyrosine	2.9 ± 0.3^Ab^	3.1 ± 0.5^b^	4.2 ± 0.6^Aa^	4.2 ± 0.4^Aa^	2.6 ± 0.2^Bc^	3.0 ± 0.2^bc^	3.1 ± 0.3^Bab^	3.3 ± 0.3^Ba^	<0.001	<0.001
Alanine	5.3 ± 0.3^B^	5.7 ± 0.7	6.1 ± 1.2	5.6 ± 0.8	6.2 ± 0.7 ^A^	5.9 ± 0.4	5.8 ± 0.3	5.6 ± 0.6	0.508	0.244
Aspartic acid	6.6 ± 0.2^Bab^	6.7 ± 0.5^a^	6.3 ± 0.7^Bb^	5.9 ± 0.8^b^	7.3 ± 0.4^Aa^	7.0 ± 0.4^ab^	7.0 ± 0.4^Aa^	6.5 ± 0.3^b^	<0.001	<0.001
Glutamic acid	8.8 ± 0.3^Ba^	9.4 ± 0.4^Ba^	9.2 ± 0.6^Ba^	7.2 ± 0.8^Bb^	10.0 ± 0.6^A^	10.2 ± 0.3^A^	9.8 ± 0.3 ^A^	10.1 ± 0.5 ^A^	<0.001	<0.001
Serine	3.8 ± 0.3^bc^	3.0 ± 0.2^Bc^	6.5 ± 0.7^Aa^	5.1 ± 2.3^ab^	3.7 ± 0.3^a^	4.5 ± 0.3^Ab^	4.8 ± 0.2^Bb^	4.6 ± 0.3^b^	<0.001	0.384
***ΣNEAA***	68.0 ± 1.3^a^	66.9 ± 1.6^a^	62.1 ± 2.4^Bb^	59.1 ± 2.3^Bc^	69.2 ± 1.5^a^	67.2 ± 1.1^b^	67.1 ± 1.5^Ab^	65.2 ± 0.5^Ac^	<0.001	<0.001
***ΣEAA/ΣNEAA***	0.47 ± 0.03^c^	0.49 ± 0.04^c^	0.61 ± 0.06^Ab^	0.69 ± 0.07^Aa^	0.45 ± 0.03^c^	0.49 ± 0.02^b^	0.49 ± 0.03^Bb^	0.53 ± 0.01^Ba^	<0.001	<0.001

*P*_FT_ and *P*_SP_ are the significant levels (GLM) for the effects of feeding time and species, respectively.

*Arginine/Glycine could not be separated

**General linear model (GLM) analysis of variance with feeding time and species as fixed factors; where significant differences were detected (*P* < 0.05), a Tukey’s pairwise comparison or a *t*-test were applied.

^a–c^Different superscript lowercase letters within each species and each total amino acid in the same row indicate significant differences (*P* < 0.05) throughout feeding time.

^A-B^Different uppercase superscript letters within each feeding time and each total amino acid in the same row indicate significant differences (*P* < 0.05) between the species at the same feeding time.

Although no significant difference was found in the percentage of ΣEAA and ΣNEAA between EC and ST (*P*_FT_ ≥ 0.108) at the pre-feeding baseline specimens, EC gonads displayed higher levels of isoleucine, leucine, valine, and tyrosine, while ST gonads contained elevated amounts of threonine, asparagine, alanine, aspartic acid, and glutamic acid. Moreover, EC gonads exhibited a lower ΣEAA/ΣNEAA ratio at pre-feeding baseline specimens, compared to previously reported values^40^, likely influenced by seasonal and geographical variations. Notably, the TAA profile of sea urchins changed during the roe enhancement, with EC gonads exhibiting a significantly higher percentage of ΣEEA compared to their ST counterparts. Furthermore, the EC fed formulated feed had a significantly higher gonads ratio of ΣEAA/ΣNEEA by the end of the roe enhancement period than that of ST (40.9 ± 2.3% vs 34.8 ± 0.5%), which could be attributed to differences in protein assimilation efficiency between the two species.

**Fig. 3:**
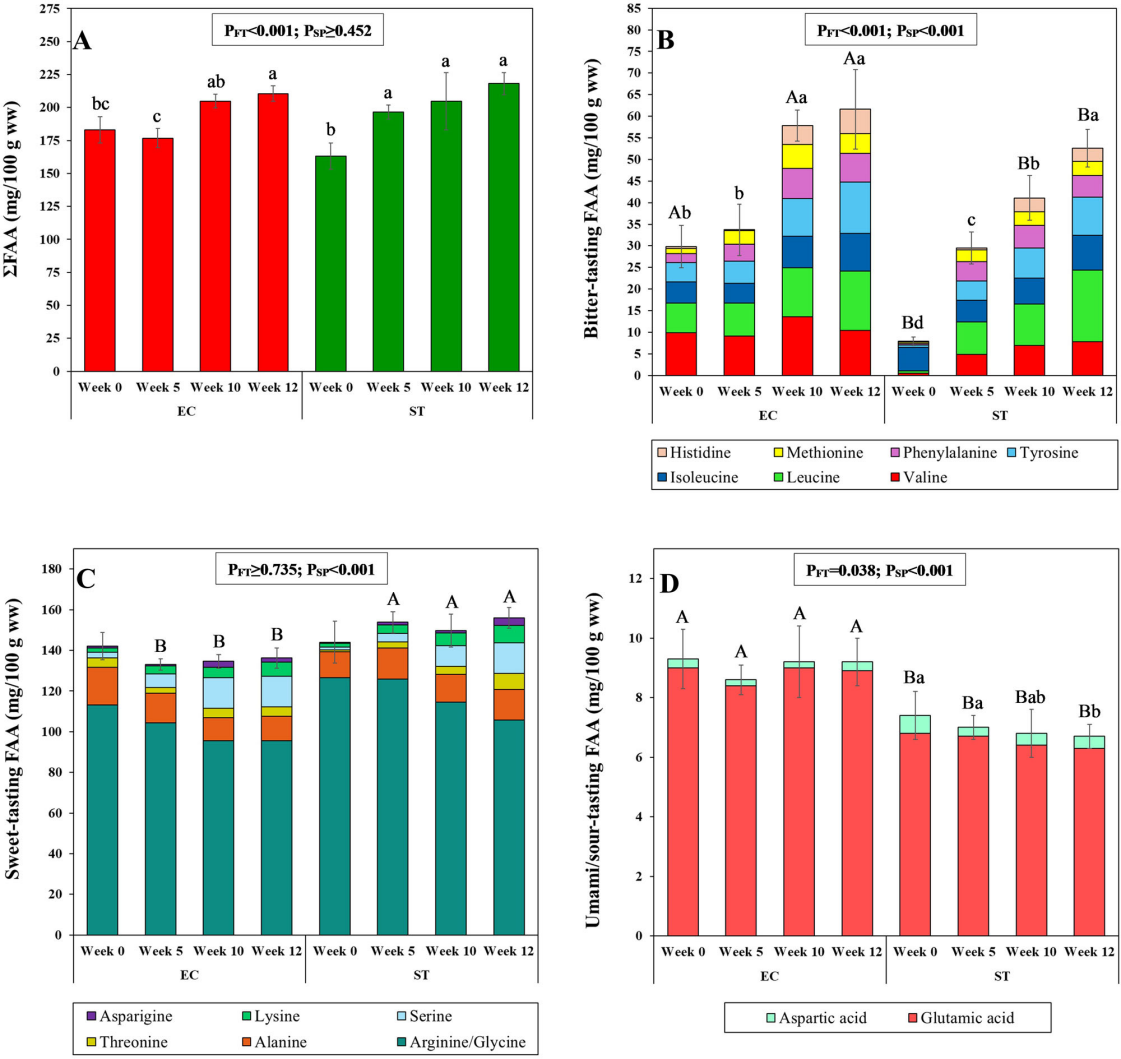
Amino acid distribution. Bar plots of the total concentration of free amino acid (ΣFAA) (**Α**) distribution of bitter-tasting FFA (**B**), sweet-tasting FAA (**C**) and umami/sour-tasting FAA (**D**) of *Echinus esculentus* (EC) and *Strongylocentrotus droebachiensis* (ST) gonads. The error bars represent one standard deviation of either the ΣFAA or the total concentration of each FFA tasting category. P_FT_ and P_SP_ are the significant levels (GLM) for the effects of feeding time and species, respectively. ^a–d^Different lowercase letters within each species indicate significant differences (*P* < 0.05) in ΣFAA, and the total concentration of bitter-tasting FAA, sweet-tasting FAA and umami/sour-tasting FAA throughout feeding time. ^A-B^Different uppercase letters within each feeding time indicate significant differences (*P* < 0.05) in ΣFAA, and the total concentration of bitter-tasting FAA, sweet-tasting FAA and umami/sour-tasting FAA between the species at the same feeding time. This graph was created with OriginPro 2022 (OriginLab Corp.).

Beyond their nutritional value, amino acids also play a crucial role in taste perception when present as FAA^13,40^. The roe enhancement significantly affected the total concentration of FAA (*P*_FT_ < 0.001), with higher average ΣFAA levels observed after the feeding (214.4 ± 10.3 mg/100 g ww) compared to the initial values of pre-feeding baseline specimens (172.6 ± 12.8 mg/100 g ww) (Fig. 3, Supplementary Table S3). Similar trends have been reported in previous studies on various sea urchin species, including ST, fed different artificial diets^9,47,48^.

Notably, both EC and ST species exhibited a significant increase in the total concentration of bitter-tasting FAA throughout roe enhancement (*P*_FT_ < 0.001), while their total amount of sweet- and umami/sour-tasting FAA remained similar to those of pre-feeding baseline specimens (*P*_FT_ ≥ 0.735 and 0.108, respectively). Moreover, a correlation was found between the bitter-tasting FAA and their corresponding bitter TAA precursors (*r* = 0.88, *P* = 0.004), suggesting that the substantial rise in the concentrations of bitter-tasting TAA seems to have positively influenced the levels of bitter-tasting amino acids in the FAA pool. Moreover, the significant rises in lysine and serine concentrations compensated for the significant reduction in arginine/glycine content during the feeding, resulting in no significant differences in the total sweet-tasting FAA amount during the feeding. However, due to the complex interactions between compounds, the overall flavour experience cannot always be accurately predicted from the FAA profile alone.

At the pre-feeding baseline, EC gonads contained significantly higher concentrations of bitter-tasting FAA than those of ST (*P*_SP_ < 0.001) (Fig. 3, Supplementary Table S3). Specifically, leucine, valine, methionine, phenylamine and tyrosine were all significantly elevated in EC. This trend persisted throughout the roe enhancement period for most bitter-tasting FAA (except leucine), with EC maintaining higher concentrations than ST. Nevertheless, the increase in bitter-tasting FAA content during the feeding period was more pronounced in ST (+44.8 mg/100 g) than in EC (+30.7 mg/100 g). Similarly, EC gonads consistently showed significantly higher levels of umami/sour-tasting FAA before and during the roe enhancement period, with glutamic acid representing the predominant compound in this taste category. In contrast, sweet-tasting FAA levels were initially comparable between pre-feeding baseline species, but EC displayed significantly lower concentrations than ST throughout the feeding period. The differing percentage changes in bitter- and umami/sour-tasting FAA between species, as well as the greater accumulation of sweet-tasting FAA in ST gonads over time, may reflect species-specific responses to the digestibility and assimilation of the artificial diet^47^.

Overall, the roe enhancement positively influenced the nutritional profile of both species, enhancing EAA levels and highlighting their potential as a good source of high-quality protein. However, inherent differences between EC and ST remained, with EC exhibiting higher concentrations of bitter- and umami/sour-tasting FAA, while ST showed a greater increase in sweet-tasting FAA, suggesting that, from a flavour perspective, EC gonads may be less appealing than the commercially accepted ST. While the amino acid profile of the feed was not included due to proprietary constraints, doing so could have allowed for correlations between diet composition and FAA changes, providing a more complete picture of the mechanisms underlying flavour development. To potentially overcome the flavour limitations of EC gonads, exploring dietary formulations with varying protein content and amino acid compositions may provide a mean to modulate FAA composition toward a more desirable balance flavour-associated FAAs, aiming for a robust umami character complemented by a pleasant sweet profile.

### Microbial quality

**Fig. 4:**
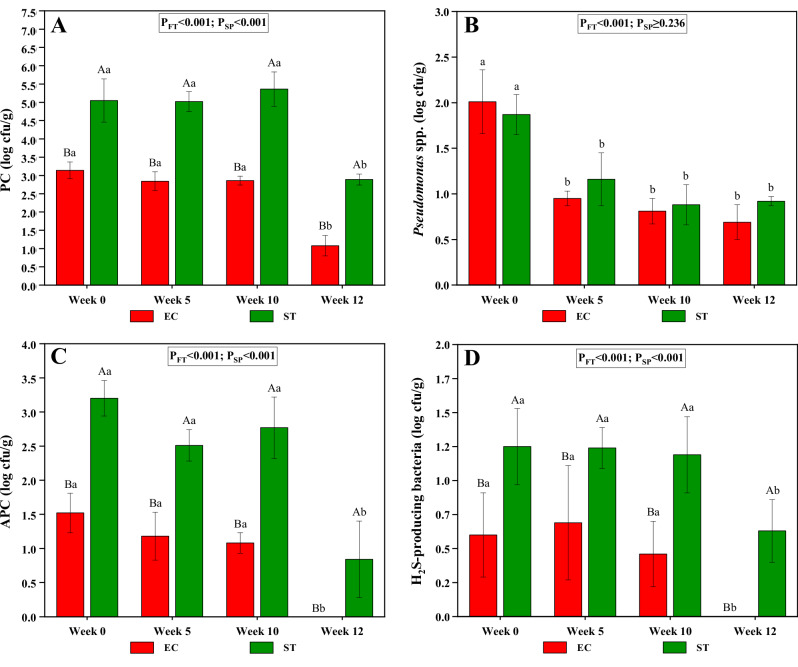
Microbial quality. Bar plots of psychrotrophic aerobic plate count (PC) (**A**), *Pseudomonas* spp. (**B**), aerobic plate count (APC) (**C**) and H_2_S-producing bacteria (**D**) of *Echinus esculentus* (EC) and *Strongylocentrotus droebachiensis* (ST) gonads. The error bars represent one standard deviation. P_FT_ and P_SP_ are the significant levels (GLM) for the effects of feeding time and species, respectively. ^a–b^Different lowercase letters within each species indicate significant differences (*P* < 0.05) in microbial counts throughout feeding time. ^A-B^Different uppercase letters within each feeding time indicate significant differences (*P* < 0.05) in microbial counts between the species at the same feeding time. This graph was created with OriginPro 2022 (OriginLab Corp.).

Although microbial enumeration is a critical factor in assessing the microbial quality and storage stability of gonads^40^, this parameter is neglected during catch-based echinoculture and is not usually assessed in commercially sold gonads. However, it is a critical factor for distributing raw gonads to distant markets, as better microbial quality extends storage stability and prolongs shelf life. The roe enhancement had a significant effect on the microbial counts (*P*_FT_ < 0.001) (Fig. 4). No significant differences in the PC, APC and H_2_S-producing bacteria counts were found from week 0 (pre-feeding baseline specimens) to week 10 of roe enhancement within each species. Following the two-week starvation period, a significant reduction in microbial loads was observed with PC and APC counts dropping by ~2 log CFU/g, and H_2_S-producing bacteria by about 1 log CFU/g. The comparable levels of APC, PC, and H_2_S-producing bacteria in the gonads until the onset of starvation suggested that the cultivation system did not significantly influence the microbial loads. In aquaculture, starvation of fish before transport or slaughter is a common practice to reduce metabolic activity, oxygen demand, and empty the gut, thereby minimizing the risk of faecal contamination during processing^49^. As the amount of faecal matter notably reduced in both the gut of sea urchins and in the surrounding water during starvation, our findings highlight the important role of starvation in reducing microbial counts in gonads.

In contrast, a significant decrease in *Pseudomonas* spp. counts were already evident from week 5 onwards when compared to week 0 (pre-feeding baseline specimens). While APC and PC provide an overview of a broad range of bacterial taxa capable of growing on general media, the enumeration of *Pseudomonas* on selective media and the decline in its counts may indicate shifts in the relative abundance of microbial taxa within the gonad microbiota during roe enhancement. However, further investigation is required to better understand the dynamics and implications of these microbial shifts.

Comparing the sea urchin species, the levels of PC, APC, and H_2_S-producing bacteria differed significantly (*P*_SP_ < 0.001) between the species, while *Pseudomonas* spp. showed no significant differences (*P*_SP_ ≥ 0.236). Prior to roe enhancement, the microbial counts of EC gonads align with those reported in a previous study on EC harvested in Mid-Norway during winter/spring^40^, while no such data are available for ST. Moreover, ST had higher microbial counts for APC, PC and H_2_S-producing bacteria than EC, both in the pre-feeding baseline specimens and throughout the roe enhancement. These differences may be attributed to size disparities. The smaller body size of ST results in their gonads being in closer contact with internal organs, increasing the likelihood of microbial contamination. Additionally, the extraction of gonads from smaller sea urchins presents technical challenges, making it difficult to avoid contact with the internal cavity, which further elevates the risk of contamination during processing. Therefore, in terms of microbial quality, EC gonads are superior to ST gonads, which, as mentioned, are considered the global commercial benchmark.

### Sensory assessment

The odorous sensory attributes (intensity, cucumber, fresh sea, off odour) of both EC and ST gonads were significantly decreased by roe enhancement (*P*_FT_ < 0.001) compared to the pre-feeding baseline specimens (Table 5). These observations are consistent with previous studies reporting reduced smell scores in sea urchins fed with artificial diets^3,32^ and further reinforces the notion that diet plays a key role in modulating odour-related sensory perceptions in the gonads.

**Table 5:** Sensory attributes score (mean ± SD) of *Echinus esculentus* (EC) and *Strongylocentrotus droebachiensis* (ST) gonads

	EC		ST		GLM^*^	
Sensory attribute	Week 0	Week 12	Week 0	Week 12	P_FT_	P_SP_
Red	2.9 ± 0.9^b^	3.1 ± 1.1^b^	7.0 ± 1.0^a^	6.7 ± 1.4^a^	0.072	<0.001
Yellow	4.7 ± 1.6^b^	4.4 ± 1.6^bc^	5.5 ± 1.9^ab^	6.3 ± 1.7^a^	0.618	<0.001
Brown	6.0 ± 1.7^a^	6.3 ± 1.9^a^	4.2 ± 2.2^b^	3.8 ± 1.7^b^	0.351	<0.001
Viscosity	6.7 ± 1.2^ab^	5.6 ± 1.5^bc^	4.9 ± 1.8^cd^	4.4 ± 1.8^d^	0.909	<0.001
Watery	5.6 ± 1.6	5.9 ± 1.8	6.3 ± 1.8	6.5 ± 1.8	0.798	0.473
Odour intensity	5.4 ± 1.2^a^	4.4 ± 1.9^b^	3.7 ± 1.4^bc^	2.9 ± 1.2^c^	<0.001	<0.001
Cucumber odour	5.9 ± 1.7^a^	4.9 ± 2.2^bc^	5.0 ± 2.2^ab^	4.2 ± 2.1^c^	<0.001	0.011
Fresh sea	6.1 ± 1.5^a^	4.4 ± 1.8^b^	4.1 ± 1.5^bc^	3.3 ± 1.4^c^	<0.001	<0.001
Off odour	3.5 ± 1.1^a^	2.8 ± 0.5^b^	2.7 ± 1.1^b^	2.0 ± 0.5^c^	<0.001	0.002
Umami	5.3 ± 1.1	4.9 ± 1.2	5.0 ± 1.4	4.7 ± 1.8	0.113	0.064
Bitter	3.9 ± 1.5^bc^	5.5 ± 2.0^a^	2.9 ± 1.4^c^	3.8 ± 2.1^bc^	0.001	<0.001
Sweet	4.4 ± 1.5^bc^	3.7 ± 1.4^c^	5.7 ± 1.4^a^	4.9 ± 1.6^b^	0.004	<0.001
Salty	4.8 ± 1.1^a^	4.8 ± 1.0^a^	3.8 ± 1.3^b^	4.4 ± 1.4^ab^	0.229	<0.001
Off taste	4.9 ± 1.6^ab^	5.3 ± 1.7^a^	3.5 ± 1.5^c^	3.9 ± 1.7^bc^	0.189	<0.001
Chewiness	6.5 ± 1.3^a^	6.8 ± 1.4^a^	4.4 ± 1.7^b^	4.1 ± 1.8^b^	0.778	<0.001
Grainy	3.7 ± 1.6	3.1 ± 1.1	3.4 ± 1.8	2.9 ± 1.6	0.910	0.396
Aftertaste	6.7 ± 1.3^a^	7.0 ± 1.4^a^	5.6 ± 1.4^b^	5.1 ± 1.6^b^	0.645	<0.001

*P*_FT_ and *P*_SP_ are the significant levels (GLM) for the effects of feeding time and species, respectively.

^*^General linear model (GLM) analysis of variance with feeding time and species as fixed factors; where significant differences were detected (*P* < 0.05), a Tukey’s pairwise comparison was applied.

^a–d^Different superscript letters within the same sensory attribute indicate significant differences (*P* < 0.05) between the samples.

A 1-9 scale and descriptive analyses with 12 semi-trained assessors were used.

Taste is a complex phenomenon involving numerous interactions, and studies on sea urchins have shown that it is closely linked to FAA, with glutamic acid, glycine, alanine, valine, methionine, arginine, lysine, and serine playing key roles as taste-active compounds^3,50^. However, the taste perception of each amino acid are influenced by factors such as concentration, pH, and the presence of enhancing or inhibiting substances^24^. In the present study, a significant increase in bitterness was recorded after the roe enhancement for both species (*P*_FT_ = 0.001), which may be attributed to the corresponding rise in bitter-tasting FAA, aligning with previous findings that associate elevated levels of valine, leucine, and isoleucine with increased bitterness during artificial feeding^24^. Supporting this, a correlation was found between the bitter-tasting FAA and the bitterness score from the sensory panel (*r* = 0.58, *P* = 0.02).

Moreover, the observed decline in sensory sweetness after roe enhancement (*P*_FT_ = 0.004) contradicts prior studies^35,37^, which often report an increase in sweetness due to higher alanine levels. In this study, this decline may be attributed to reduced levels of arginine/glycine, along with relatively low alanine concentrations. This conclusion is further supported by evidence showing that the omission or reduction of glycine and alanine leads to pronounced bitterness^50^, a finding consistent with our results. Meanwhile, the stability of umami perception (*P*_FT_ ≥ 0.113) aligns with the unchanged levels of umami-tasting FAA, indicating a consistent flavour profile in this aspect.

Sensory panel assessments and instrumental colour measurements confirmed that the formulated feed did not alter colour perception (*P*_FT_ ≥ 0.072) relative to pre-feeding baseline specimens. However, sensory assessment contradicted instrumental texture analysis as the panel detected no significant changes in chewiness and graininess (*P*_FT_ ≥ 0.778 and 0.910, respectively). While some studies^11,17^ reported similar findings, others found artificial diets had varying effects^3,51^, pinpointing the role of feed type and its proximate composition in the final textural perception. Nevertheless, our results highlight the importance of sensory evaluation in complementing instrumental analysis, as instrumental measurements provide valuable insights but may not fully capture the complexities of human sensory perception.

The organoleptic characteristics of sea urchin gonads, including appearance, taste, flavour, and odour, are considered species-specific^24^ and influenced by genetics, environmental factors, and food availability^52^. In this study, clear species-related differences were observed between EC and ST. This species-specific variation may explain the stronger odour intensity, the fresh sea odour and off-odour in EC compared to commercially well-established ST (*P*_SP_ ≤ 0.002), observed both in the pre-feeding baseline specimens and following roe enhancement.

The sensory panel detected higher bitterness and saltiness in EC gonads compared to their ST counterparts, both at the pre-feeding baseline specimens and after roe enhancement (*P*_SP_ < 0.001), while ST gonads after roe enhancement were rated significantly sweeter than those of EC (*P*_SP_ < 0.001). Additionally, EC sea urchins exhibited higher off-taste and aftertaste scores than corresponding ST gonads after the roe enhancement period (*P*_SP_ < 0.001). These sensory differences are likely linked to the chemical composition of the gonads, as EC exhibited higher concentrations of bitter-tasting FAA, which may have suppressed sweetness due to the interaction between these tastes in the sensory palette. The correlation between bitterness and both off-taste (*r* = 0.61, *P* < 0.001) and aftertaste (*r* = 0.24, *P* < 0.001) further supports the role of FAA composition in shaping the sensory profile of EC gonads.

Both prior and after roe enhancement, EC gonads were consistently rated as less reddish, more yellowish and more brownish than ST specimens (*P*_SP_ < 0.001), which aligns with instrumental colour analysis. Additionally, the higher chewiness scores for EC gonads(*P*_SP_ < 0.001), supported by instrumental texture measurements, are in agreement with previous studies reporting that larger gonads typically tend to exhibit greater sensory firmness compared to smaller ones^15^.

**Fig. 5:**
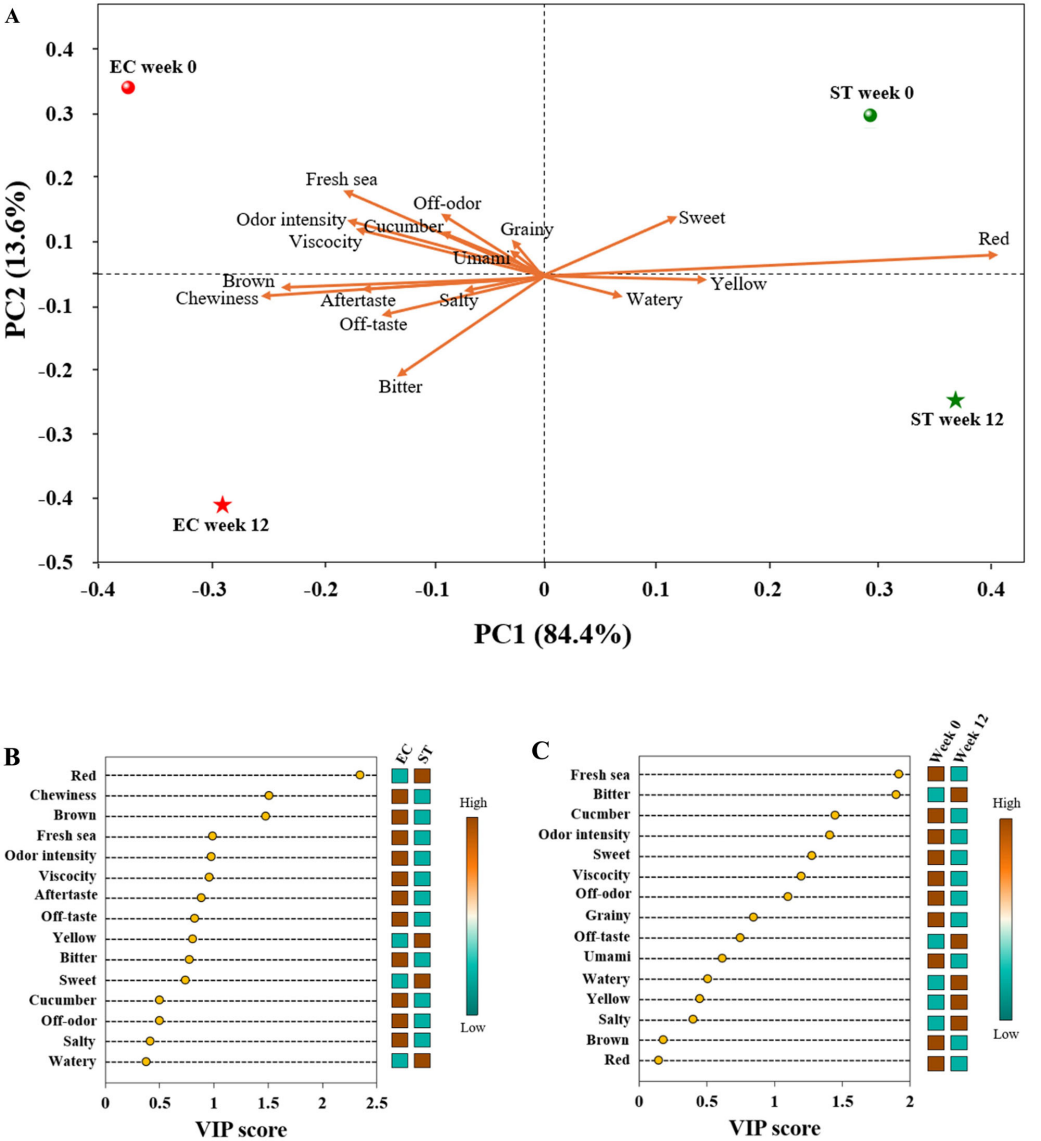
Sensory attributes. Partial least squares discriminant analysis (PLS-DA) biplot of sensory attributes of *Echinus esculentus* (EC) and *Strongylocentrotus droebachiensis* (ST) gonads (**A**). Variable importance in projection (VIP) plots (**B**, **C**) showing key sensory attributes (VIP > 1) which importantly contribute to differences in sensory profile between the feeding time and species, respectively. The relative sensory score of the key sensory attributes per feeding time and species were displayed by the colour boxes, with brown and dark teal indicating high and low relative sensory score, respectively. This graph was created with OriginPro 2022 (OriginLab Corp.).

To further summarize the impact of feeding and species on the sensory attributes, a PLS-DA biplot was conducted (Fig. 5). The analysis highlighted key sensory attributes that discriminated between the groups, with variables having a VIP score greater than 1 identified as significant contributors to sensory differentiation (Fig. 5B, C). The sensory profile of sea urchin gonads from the pre-feeding baseline specimens was distinctly different from that of urchins fed formulated feed for both EC and ST. Key sensory attributes driving this differentiation included fresh sea odour, cucumber odour, off-odour, odour intensity, viscosity, as well as sweet and bitter taste, indicating a perceptual shift in sensory perception over the roe enhancement. Additionally, the PLS-DA analysis effectively distinguished ST from EC samples, with reddish and brownish coloration, chewiness, viscosity, fresh sea odour, and odour intensity identified as the primary factors differentiating the sensory profiles of these species. The persistence of inherent differences in sensory profiles was anticipated, given that the limited duration of the feeding trial is unlikely to substantially modify species-specific biochemical and organoleptic traits, with the ST commercially accepted reference standard maintaining a more desirable flavour.

## Methods

### Sea urchin echinoculture system

Six tanks of 850 l each were set up and filled with filtered seawater (1μm) supplied through an inshore sub-sea pipeline in the Trondheimfjorden (Norway), collecting seawater from about 70 m depth. A flow-through system was used, and the seawater flow rate was kept at 2 l/min. The seawater temperature was set to 10 °C and continuously monitored in the inflow water using an EBI-125A temperature logger. Salinity, dissolved oxygen and pH were measured daily in each tank using a portable multi-parameter metre (Hach HQ40d multi–Portable Metre, Hach, USA) equipped with a chloride electrode (Hach Intellical™ ISECL181, Hach, CO, USA), an optical dissolved oxygen sensor (Hach Intellical™ LDO101, Hach, CO, USA) and a pH electrode (Hach Intellical™ PHC108, Hach, CO, USA). The salinity, dissolved oxygen and pH remained constant at 34.89 ± 0.1‰, 97.1 ± 0.9% and 8.1 ± 0.1, respectively, throughout the roe enhancement period. The levels of ammonia and nitrite were determined photometrically every second day, using an ammonium test (Ref 1.14752.0001, Merch) and nitrite test (1.14776.001, Merck). Ammonia and nitrite levels were below the methods’ detection limit (0.016 mg/l NH_3_ and 0.007 mg/l NO_2_-, respectively). The sea urchins were exposed to a simulated natural photoperiod corresponding to Trondheim latitude (63.4468°N) from end of February to the mid-May 2023. Maximum light intensity at the water surface was approximately 150 lux.

### Experimental design, feeding trial and sampling plan

**Fig. 6:**
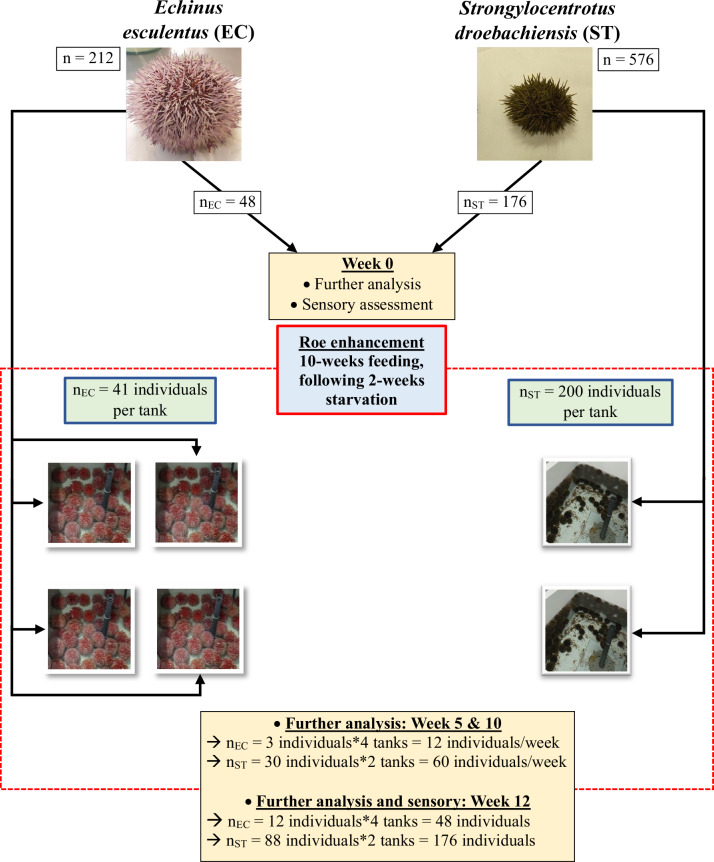
Experimental overview. A part of the sea urchins was used for physicochemical, biochemical, and microbial analysis, as well as sensory assessment, prior to roe enhancement. The remaining individuals were fed formulated feed for 10 weeks, following by 2 weeks starvation. Subsets were sampled at weeks 5 and 10 for physicochemical, biochemical, and microbial analysis, while week 12 included these analyses along with sensory assessment. Photos of sea urchins and tanks were taken by us.

A total of 212 *Echinus esculentus* (EC) and 576 *Strongylocentrotus droebachiensis* (ST) individuals were randomly harvested from Bjugn municipality (Mid-Norway; 63.7874°N, 9.5581°E) on 27 February 2023, nearly at the onset of the spring season. The sea urchins were transferred to NTNU Centre of Fisheries and Aquaculture (SeaLab) (Trondheim, Norway) in isothermal transport boxes with natural seawater. Α sample of the sea urchins was used to evaluate the physicochemical attributes, biochemical composition, microbial quality and sensory attributes of sea urchins at the time of harvest (initial census) (*n*_EC_ = 48 and *n*_ST_ = 176) (Fig. 6), providing a biologically relevant baseline that reflects the natural gonad status prior to experimental intervention. The remaining EC sea urchins were distributed in four tanks (*n*_EC_ = 41 individuals per tank) while ST sea urchins were distributed in two tanks (*n*_ST_ = 200 individuals per tank). During the experimental trial, sea urchins were fed the Urchinomics Urchin Feed V10.1.10, a formulated algae-based diet composed primarily of human-grade offcuts from wild-harvested and farmed *Saccharina japonica* (kombu)^16^. The feed was originally developed by Nofima over the past two decades and has proven effective in several feeding trials. It is now licensed to Urchinomics and commercially produced by Mitsubishi Corporation for large-scale applications. The proximate composition of the feed was determined through our own laboratory analyses as follows: water (12.8 ± 0.1%), ash (18.9 ± 0.2%), protein (14.9 ± 0.2%), lipids (1.7 ± 0.1%) and carbohydrates (51.2 ± 0.2%). As is common in studies involving non-commercially formulated feeds^16,26^, proprietary considerations and confidentiality agreements with the supplier limit the disclosure of full compositional details. This feed has been successfully applied in previous studies with *S. droebachiensis*^26,53^ and other sea urchin species^16^, providing a reliable benchmark against which the performance of *E. esculentus* can be evaluated. The sea urchins were fed ad libitum (feed was provided at a surplus for the duration of the trial), with the amount of feed offered recorded daily. Uneaten feed and faeces were removed daily and their quantities recorded each day. The tanks were cleaned twice a week by flushing the bottom. The roe enhancement experiment lasted for 12 weeks, ending on May the 15^th^ 2023. At weeks 5 and 10, twelve EC (3 individuals per tank) and sixty ST (30 individuals per tank) were randomly removed from the tanks and analysed. Sea urchins were kept in the tanks for two more weeks (until week 12) under starvation to allow gut evacuation. The starved sea urchins were subsequently removed from the tanks and subjected to further analysis and sensory assessment. A specific sampling plan was followed for each investigated parameter (Table 6).

**Table 6:** The sampling plan followed for each investigated parameter

Sampling week					
Parameter	Species	Week 0	Week 5	Week 10	Week 12
Biometrics (WD, HD, TBWW)	EC	212	12	12	140
	ST	576	60	60	280
Biometrics (GWW, GWWC, GI)	EC	48	12	12	130
	ST	176	60	60	260
GPE	EC	–	12	12	–
	ST	–	60	60	–
Colour	EC	48	12	12	130
	ST	176	60	60	280
Texture	EC	24	12	12	24
	ST	48	24	24	48
pH	EC	12	12	12	12
	ST	12	12	12	12
Water & ash content	EC	12	12	12	12
	ST	12	12	12	12
Protein & lipid content	EC	8	8	8	8
	ST	8	8	8	8
Fatty acids, TAAs & FAAs distribution	EC	8	8	8	8
	ST	8	8	8	8
Microbial counts	EC	4	4	4	4
	ST	4	4	4	4
Sensory assessment	EC	30	–	–	30
	ST	100	–	–	100

The numbers represent the amounts of conducted measurements per species (EC: *Echinus esculentus* and ST: *Strongylocentrotus droebachiensis*) and sampling week.

*WD* Width diameter, *HD* Height diameter, *TBWW* Total body wet weight, *GWW* Gonad wet weight, *GWWC* Gonad wet weight change, *GI* Gonad index, *GPE* Gonad production efficiency, *TAA* Total amino acids, *FAA* Free amino acids

### Survival rate and production efficiency

Survival rate (%) was estimated by comparing the number of live sea urchins at the start of the experiment with the number of individuals that die during the roe enhancement period, as described in formula (1).

1 $${\rm{Survival}}\, {\rm{rate}}\left( \% \right)=\frac{{\rm{Number}}\, {\rm{of}}\,{\rm{live}}\, {\rm{individuals}}\, {\rm{at}}\, {\rm{the}}\, {\rm{end}}\, {\rm{of}}\, {\rm{roe}}\, {\rm{enhancement}}}{{\rm{Number}}\, {\rm{of}}\, {\rm{live}}\, {\rm{individuals}}\, {\rm{at}}\, {\rm{the}}\, {\rm{start}}\, {\rm{of}}\, {\rm{roe}}\, {\rm{enhancement}}}\times 100$$

### Biometric parameters and gonad production efficiency

The total body wet weight (TBWW) (g), height (HD) and width (WD) diameters (mm) of all individuals (*n*_EC_ = 212 and *n*_ST_ = 576) were measured when the sea urchins arrived at the lab facilities. Moreover, 48 EC and 176 ST specimens were dissected, and their gonads were removed, damp-dried and weighed to be used as a reference for the catch before roe enhancement. According to the sampling plan, the same procedure was followed during the roe enhancement of sea urchins (Table 6). Moreover, the gonad wet weight (GWW) was measured, as well as the gonad wet weight change (GWWC) (%) and the gonad index (GI) (%) were calculated using formulas (2) and (3), respectively.

2 $${\rm{GWWC}}\,\left( \% \right)=\frac{{\rm{GWW}}\,\left({\rm{g}}\right)-{\rm{Initial}}\, {\rm{GWW}}\,({\rm{g}})}{{\rm{Initial}}\,{\rm{GWW}}\,({\rm{g}})}\times 100$$

3 $${\rm{GI}}\,\left( \% \right)=\frac{{\rm{GWW}}\,\left({\rm{g}}\right)}{{\rm{TBWW}}\,({\rm{g}})}\times 100$$

In addition, the gonad production efficiency (GPE) (%) was assessed as the increase in gonad dry weight (GDW) relative to the dry feed consumed (DFC), as described in formula (4). In this study, GPE was calculated for two time intervals, week 0–5 and week 5–10, to monitor the efficiency of feed utilization for gonad growth during the two time intervals.

4 $${\rm{GPE}}\,\left( \% \right)=\frac{{\rm{Final}}\, {\rm{GDW}}\,\left({\rm{g}}\right)-{\rm{Initial}}\,{\rm{GDW}}\,({\rm{g}})}{\rm{DFC}\,({\rm{g}})}\times 100$$

### Colorimetric parameters

The gonads’ colorimetric parameters were measured on a DigiEye full system (VeriVide Ltd., Leicester, UK) according to the sampling plan (Table 6). Five gonads from each dissected specimen were placed in a standardised light-box (daylight, 6400 K) and photographed using a digital camera (Nikon D80, 35 mm lens, Nikon Corp., Japan). The software DigiPix (version 2.8) was used to calculate L* (lightness), a* (redness) and b* (yellowness) values for each gonad. Chroma (C*) and hue angle (h*) were determined using formulas (5) and (6), respectively.

5 $${\text{C}}^{* }={\left({\text{a}}^{{* }^{2}}+{\text{b}}^{{* }^{2}}\right)}^{1/2}$$

6 $${\text{h}}^{* }=\arctan \left(\frac{{\text{b}}^{* }}{{\text{a}}^{* }}\right)$$

The mean value of the five gonads was used to express the average colorimetric parameters for each specimen.

### Texture parameters

Gonad texture was analysed using a Texture Analyser TA-XT plus (Stable Micro Systems Ltd, England) equipped with a 5-kg load cell and a flat-ended cylindrical probe (12.7 mm P/0.5) as described by Tsoukalas et al.^40^. The parameters determined from the force–time curves were hardness (N), cohesiveness (dimensionless), springiness (mm), and chewiness (hardness × cohesiveness × springiness) (mJ).

### Gonad pH

The gonad pH of the sea urchins was measured on each sampling week (Table 6) using a portable multi-parameter metre (Hach HQ40d multi–Portable Metre, Hach, USA) equipped with a puncture pH electrode (Hach Intellical™ PHC108, Hach, CO, USA).

### Proximate composition

The water content of the gonads was measured by drying the samples at 105 °C for 24 h. After weighing the dried samples, they were subsequently incinerated at 500 °C for 20 h to assess the ash content. Total crude protein (%) was analysed using Kjeldahl method^54^, following the procedure outlined by Kendler et al.^55^ Total lipid extraction and quantification of total lipids were carried out using the method of Bligh and Dyer^56^. A portion of the extracted lipids was preserved in the chloroform phase at −40 °C for later fatty acid analysis.

### Fatty acid distribution

A volume of approximately 100 mg of extracted lipids was used to prepare fatty acid methyl esters (FAMEs). Initially, the extracted lipids were subjected to nitrogen evaporation until all chloroform was removed from the samples. Afterwards, FAMEs were prepared according to Metcalfe et al.^57^

The FAMEs analysis followed the procedure described by Hamed et al.^28^ and was performed using an Agilent 6850 gas chromatograph (Agilent Technologies, USA). The system featured a polyethylene glycol column (HP-INNOWAX, 250 μm inner diameter, 0.25 μm film thickness), a flame ionization detector (FID) set to 310 °C, and an injector set to 260 °C, operating at 18.1 psi with a 50:1 split ratio. The oven program was set to a constant temperature of 160 °C for 3 min, with an increase of 3 °C/min to 240 °C and held for 3 min. Fatty acids were identified by comparing the relative retention times (RRTs) of sample peaks to those of a reference standard containing 37 FAME components (Supelco 37 Component FAME Mix, Merck Life Sciences AS, Oslo, Norway). The relative abundance of each fatty acid was calculated as the ratio of its peak intensity to the total intensity of all FAME peaks in the sample.

### Total amino acids and free amino acids distribution

The total amino acids (TAAs) extraction, adapted from Blackburn^58^, allowed for the identification of 17 amino acids, both essential and non-essential. Before analysis, the samples were freeze-dried at −40 °C under a pressure of 13.3 Pa for 26 h. Freeze-dried samples (~50 mg protein) were hydrolysed with 1 ml of hydrochloric acid (6 mol/l) at 105 °C for 22 h. After hydrolysis, the solution was neutralized using sodium hydroxide. The free amino acids (FAAs) extraction was performed according to the method of Osnes and Mohr^59^. Samples were homogenized in deionized water and centrifuged at 500 × *g* for 3 min at 4 °C to isolate the soluble protein extract. The extract was mixed with 0.25 ml of sulphosalicylic acid (100 ml/l), centrifuged at 2700 × *g* for 10 min at 4 °C, and the supernatant was used for further analysis. Both the extracted solutions of TAAs and FAAs were filtered prior to analysis on an ultra-HPLC system (UltiMate 300, Thermo Scientific). The HPLC system included a Nova-Pak C18 column (WAT086344, particle size: 4 μm, dimensions: 3.9 mm × 150 mm, Waters Corp., USA), a TSP P400 pump, an UltiMate 3000WP injector, and an RF2000 detector.

The identification and quantification of TAAs and FAAs followed the procedure detailed by Kendler et al.^55^ Amino acid standards, purchased from Fluka (Switzerland), were used to compare retention times and determine concentrations. Some amino acids were excluded from the analysis: cysteine, due to its instability under acid hydrolysis conditions; proline, because it could not be detected following o-phthalaldehyde (OPA) derivatization; and tryptophan, which was degraded during acid hydrolysis. Additionally, glycine and arginine were found to co-elute during the HPLC process.

### Microbial analysis

A 10-g sample of gonads was aseptically placed in a sterile stomacher bag and diluted 1:10 with sterile peptone water, which consisted of 1.0 g bacteriological peptone (Lyngby, Oxoid, Oslo, Norway) and 8.5 g/L NaCl (AnalaR NORMAPUR® ACS). The sample was homogenised for 1 min using a Stomacher 400 Lab Blender (Seward Medical Ltd., UK). Afterwards, serial dilutions were prepared in sterile peptone water for plating. Psychrotrophic aerobic plate count (PC) was counted on Long and Hammer agar (LH) containing 1% NaCl to support the growth of marine bacteria^60^, with plates incubated at 15 °C for six days. Aerobic bacteria (APC) and H_2_S-producing bacteria were quantified using iron agar (Oxoid) enriched with 0.04% l-cysteine (Sigma-Aldrich, Oslo, Norway), where total and black colonies were counted after incubation at 22 °C for 72 h. Pseudomonas agar base (CM0559, Oxoid) with Pseudomonas CFC selective supplement SR0103 (Oxoid) was used to quantify *Pseudomonas* spp., with the plates incubated at 25 °C for 48 h.

### Sensory assessment

The sensory attributes of gonads at the pre-feeding baseline specimens (week 0) and those after roe enhancement (week 12) were assessed. Initially, gonads were carefully removed from dissected sea urchins and sequentially soaked in clean sterile artificial seawater (35‰) for 10 min and 40 min. Afterwards, the gonads were placed on a paper towel and dried for 2 h. Finally, gonads were carefully placed in plastic bags, frozen by immersion in dry ice and stored at −80 °C until the sensory assessment. Gonads were thawed at room temperature for 4 h before the sensory evaluation.

A semi-trained panel consisting of 12 assessors (6 male/6 female, aged between 20 and 55 y), employed at NTNU, performed a sensory descriptive analysis (DA) in triplicates according to the “Generic Descriptive Analysis” described by Lawless and Heymann^61^ and the ISO standard 13299^62^. The assessors were tested and trained according to ISO standard 8586^63^. The sensory laboratory follows the practice of ISO standard 8589^64^. The samples were served to assessors at room temperature in translucent plastic beakers, blind-labelled with three-digit random codes. During the session, each assessor evaluated one gonad from each of the four samples. A reference sample was also supplied to serve as a sensory benchmark for comparison with the other samples. Panelists were provided with a plastic spittoon, a glass of natural water and unsalted crackers. The panelists assessed the sensory attributes of gonads based on 17 defined descriptive terms (Table 7) using a 9-point scale, ranging from 1 - “low intensity” to 9 - “high intensity”. The attribute descriptions were developed by the semi-trained panel (*n* = 12), prior to descriptive analysis.

**Table 7:** Attribute description of *Echinus esculentus* (EC) and *Strongylocentrotus droebachiensis* (ST) gonads developed by the semi-trained panel (*n* = 12), prior to descriptive analysis

Red	Intensity of red colour
	Low intensity = not red
	High intensity = very red
Yellow	Intensity yellow colour
	Low intensity = not yellow
	High intensity = very yellow
Brown	Intensity of brown colour
	Low intensity = not brown
	High intensity = very brown
Viscosity	Thickness of liquid (sliminess)
	Low intensity = not thick, like water
	High intensity = very thick, slimy
Watery	How much the roe glistens (not including liquid around the roe).
	Low intensity = the surface looks dry
	High intensity = the surface seems wet
Odour intensity	How strong the odour is
	Low intensity = low odour
	High intensity = intense odour
Cucumber (odour)	How strong is the cucumber odour
	Low intensity = low odour
	High intensity = intense odour
Fresh sea (odour)	Intensity of seafood odour (Briny, fishy, mussels)
	Low intensity = no seafood smell
	High intensity = intense seafood smell
Off-odour	Intensity of unpleasant odour
	Low intensity = low off-odour
	High intensity = intense off-odour
Umami	Savory, meaty
	Low intensity = no umami flavour
	High intensity = intense umami flavour
Bitter	Low intensity = not bitter
	High intensity = intense bitterness
Sweet	Low intensity = not sweet
	High intensity = intense sweetness
Salty	Low intensity = no salt taste
	High intensity = intense salt taste
Off-taste	Intensity of unpleasant taste
	Low intensity = low off-taste
	High intensity = intense off-taste
Chewiness	Rubbery, gum-like
	Low intensity = not elastic, feels like it stays deformed
	High intensity = very elastic, springs back to original shape
Grainy (mouth feel)	How well can the granules be discerned. Tongue, caviar
	Low intensity = the granules are not discernable
	High intensity = the granules are clearly discernable (reference tobici roe, the roe usually used in sushi)
Aftertaste intensity	Intensity of aftertaste
	Low intensity = no aftertaste
	High intensity = distinct aftertaste

### Statistical analysis

The experimental unit for statistical analyses was the tank, with each tank treated as a replicate to account for the shared environment of sea urchins within it. For EC, four tanks were used as replicates, while for ST, two tanks were used. Sea urchins within the same tank were considered subsamples rather than independent replicates, and statistical analyses were performed using tank-level data to avoid pseudoreplication. The statistical analysis was performed by a general linear model (GLM) in IBM SPSS statistics software (release 28, IBM Corporation, USA), with feeding time and species as fixed factors and tank included as a fixed blocking factor to account for potential tank effects. After testing, no significant tank effect was detected for any of the measured parameters, so it was excluded from the final model to simplify the analysis without compromising the analysis. Statistical analysis of microbial counts was done on log-transformed data. All statistical analysis dealing with sensory assessment data was analysed using the statistical tool EyeOpenR® available in the EyeQuestion® software (EyeQuestion®, Version 4.11.61, Gelderland, Elst, The Netherlands). A partial least squares discriminant analysis (PLS-DA) with a variable importance in projection (VIP) plots was used to identify the sensory attributes that distinguish samples. Sensory attributes with a VIP > 1 were considered to be key factors in differentiating sensory perceptions. All graphs were created using OriginPro 2022 (Origin Lab Corp.). Normality tests were assessed based on the normality probability plots and homogeneity of variance was examined using Levene’s test. A Tukey’s pairwise comparison test and a *t*-test were carried out to investigate the differences between feeding time and species, respectively. Statistical differences were reported at the level of α < 0.05. All results are presented as mean values ± standard deviation (SD).

## Supplementary information

Supplementary Information

## Data Availability

The data that support the findings of this study are available from the corresponding author, upon reasonable request.

## Code availability

No custom code was used. All analyses were performed using standard software packages as described in the Methods section.
