# Supplementary material for: Impact of roe enhancement on quality parameters in sea urchins Echinus esculentus and Strongylocentrotus droebachiensis
Source: NPJ Sci Food. 2025 Nov 4;9:218. doi: 10.1038/s41538-025-00579-5 (PMC12586614; doi:10.1038/s41538-025-00579-5)
Supplement: Supplementary file 1 — Supplementary Information [file 41538_2025_579_MOESM1_ESM.docx]

**Supplementary Table S1:** Width diameter (WD), height diameter (HD), total body wet weight (TBWW), gonad wet weight (GWW), gonad production efficiency (GPE) and gonad index (GI) (mean±SD) of *Echinus esculentus* (EC) and *Strongylocentrotus droebachiensis* (ST) gonads

| **Species** | **Feeding time** | **WD (mm)** | **HD (mm)** | **TBWW (g)** | **GWW (g)** | **GPE (%)** | **GI (%)** |
| --- | --- | --- | --- | --- | --- | --- | --- |
| EC | Week 0 | 109.1±8.7^A^ | 80.4±7.9^A^ | 543.2±133.8^A^ | 35.2±13.7^A^ | 0 | 8.4±6.5 |
|  | Week 5 | 109.4±3.3^A^ | 81.4±29.9^A^ | 593.1±44.4^A^ | 41.2±16.2^A^ | 11.6±5.8^Ba^ | 7.0±2.8^B^ |
|  | Week 10 | 109.8±7.5^A^ | 81.1±6.5^A^ | 566.1±100.7^A^ | 42.0±17.3^A^ | 5.9±2.2^Bb^ | 8.0±3.5^B^ |
|  | Week 12 | 105.9±8.2^A^ | 79.4±7.9^A^ | 566.9±124.4^A^ | 41.2±16.8^A^ |  | 7.4±3.0^B^ |
|  |  |  |  |  |  |  |  |
| ST | Week 0 | 50.6±8.0^B^ | 29.1±5.5^B^ | 47.2±17.9^B^ | 2.9±2.3^Bc^ | 0 | 6.5±4.0^c^ |
|  | Week 5 | 52.8±4.3^B^ | 29.9±2.9^B^ | 52.2±4.3^B^ | 7.0±2.4^Bb^ | 22.9±1.7^Aa^ | 13.3±4.4^Ab^ |
|  | Week 10 | 49.9±7.1^B^ | 29.8±3.3^B^ | 54.3±12.9^B^ | 9.0±4.1^Ba^ | 14.8±1.3^Ab^ | 17.4±9.2^Aa^ |
|  | Week 12 | 50.0±6.4^B^ | 28.1±4.2^B^ | 52.6±15.2^B^ | 9.3±3.4^Ba^ |  | 17.7±6.7^Aa^ |
|  |  |  |  |  |  |  |  |
| GLM* | P_FT_ | 0.529 | 0.780 | 0.382 | <0.001 | <0.001 | <0.001 |
|  | P_SP_ | <0.001 | <0.001 | <0.001 | <0.001 | 0.033 | <0.001 |

*General linear model (GLM) analysis of variance with feeding time and species as fixed factors; where significant differences were detected (P<0.05), a Tukey's pairwise comparison or a t-test were applied.

P_FT_ and P_SP_ are the significant levels (GLM) for the effects of feeding time and species, respectively.

^a–c^Different superscript lowercase letters within each species and each parameter indicate significant differences (P<0.05) throughout feeding time.

^A-B^Different superscript uppercase letters within each feeding time and each parameter indicate significant differences (P<0.05) between the species at the same feeding time.

**Supplementary Table S2:** Lightness (L*), redness (a*) yellowness (b*), Chroma (C*) and hue angle (h*) (mean±SD) of *Echinus esculentus* (EC) and *Strongylocentrotus droebachiensis* (ST) gonads

| **Species** | **Feeding time** | **L*** | **a*** | **b*** | **C*** | **h*** |
| --- | --- | --- | --- | --- | --- | --- |
| EC | Week 0 | 56.3±5.1 | 11.7±1.9^B^ | 29.2±3.7^B^ | 31.5±3.9^B^ | 68.1±2.0^A^ |
|  | Week 5 | 55.4±5.4 | 10.3±2.3^B^ | 27.1±5.2^B^ | 28.9±5.6^B^ | 69.3±1.9^A^ |
|  | Week 10 | 51.6±5.7^B^ | 14.1±2.0^B^ | 29.8±7.3^B^ | 33.1±7.4^B^ | 63.9±3.2 |
|  | Week 12 | 52.1±5.8^B^ | 11.6±2.5^B^ | 28.3±5.2^B^ | 30.6±5.5^B^ | 67.7±3.0^A^ |
|  |  |  |  |  |  |  |
| ST | Week 0 | 53.9±3.4 | 25.6±2.9^A^ | 43.0±5.1^A^ | 50.1±5.3^A^ | 59.2±3.1^B^ |
|  | Week 5 | 54.7±3.8 | 23.6±4.6^A^ | 43.2±7.7^A^ | 49.3±8.6^A^ | 61.3±3.1^B^ |
|  | Week 10 | 59.9±3.4^A^ | 21.2±2.3^A^ | 42.6±9.5^A^ | 47.7±9.1^A^ | 62.8±4.3 |
|  | Week 12 | 60.1±4.5^A^ | 24.6±4.3^A^ | 46.0±5.2^A^ | 52.2±9.3^A^ | 61.7±3.3^B^ |
|  |  |  |  |  |  |  |
| GLM** | P_FT_ | 0.105 | 0.252 | 0.438 | 0.434 | 0.163 |
|  | P_SP_ | <0.001 | <0.001 | <0.001 | <0.001 | <0.001 |

**General linear model (GLM) analysis of variance with feeding time and species as fixed factors; where significant differences were detected (P<0.05), a t-test were applied.

P_FT_ and P_SP_ are the significant levels (GLM) for the effects of feeding time and species, respectively.

^A-B^Different superscript uppercase letters within each feeding time and each parameter indicate significant differences (P < 0.05) between the species at the same feeding time.

**Supplementary Table S3:** Free animo acids (FAA) (mg/100g ww) distribution (mean±SD) of *Echinus esculentus* (EC) and *Strongylocentrotus droebachiensis* (ST) gonads

|  | **EC** |  |  |  | **SP** |  |  |  | **GLM**** | |
| --- | --- | --- | --- | --- | --- | --- | --- | --- | --- | --- |
| **Amino acids** | **Week 0** | **Week 5** | **Week 10** | **Week 12** | **Week 0** | **Week 5** | **Week 10** | **Week 12** | **P_FT_** | **P_SP_** |
| **Bitter** |  |  |  |  |  |  |  |  |  |  |
| Histidine | 0.4±0.1^Ab^ | 0.2±0.1^Bb^ | 4.3±2.2^a^ | 5.6±1.3^Aa^ | 0.2±0.1^Bb^ | 0.4±0.1^Ab^ | 3.2±0.5^a^ | 3.1±0.5^Ba^ | <0.001 | 0.016 |
| Isoleucine | 4.9±1.8^b^ | 4.5±1.5^b^ | 7.3±1.5^ab^ | 8.7±1.7^a^ | 5.4±0.1^ab^ | 5.0±1.1^b^ | 6.0±0.7^ab^ | 8.0±2.5^a^ | <0.001 | 0.338 |
| Leucine | 6.8±2.1^Ab^ | 7.7±1.7^b^ | 11.3±2.1^ab^ | 13.8±2.7^a^ | 0.6±0.2^Bc^ | 7.5±1.3^b^ | 9.6±1.2^b^ | 16.6±3.4^a^ | <0.001 | 0.019 |
| Methionine | 1.2±0.8^Ac^ | 3.1±0.7^b^ | 5.6±0.9^Aa^ | 4.6±0.6^Aab^ | 0.3±0.1^Bb^ | 2.8±0.5^a^ | 3.2±0.5^Ba^ | 3.2±0.4^Ba^ | <0.001 | <0.001 |
| Phenylalanine | 2.1±0.5^Ab^ | 4.0±0.9^b^ | 7.0±0.9^Aa^ | 6.6±0.6^Aa^ | 0.4±0.1^Bb^ | 4.4±0.6^a^ | 5.2±0.7^Ba^ | 5.0±0.4^Ba^ | <0.001 | <0.001 |
| Valine | 9.9±1.4^Aab^ | 9.1±1.2^Ab^ | 13.6±2.1^Aa^ | 10.4±1.8^Aab^ | 0.5±0.2^Bc^ | 4.9±0.5^Bb^ | 6.9±0.7^Ba^ | 7.8±0.6^Ba^ | <0.001 | <0.001 |
| Tyrosine | 4.5±0.7^Ac^ | 5.1±0.5^c^ | 8.7±0.9^b^ | 11.9±0.7^Aa^ | 0.5±1.2^Bd^ | 4.5±0.9^c^ | 7.0±1.1^b^ | 8.9±0.6^Ba^ | <0.001 | <0.001 |
| *ΣBitter* | 30.8±4.6^Ab^ | 33.6±5.4^b^ | 57.8±3.6^Aa^ | 61.5±9.2^Aa^ | 7.8±1.0^Bd^ | 29.6±3.7^c^ | 41.1±5.2^Bb^ | 52.6±4.4^Ba^ | <0.001 | <0.001 |
|  |  |  |  |  |  |  |  |  |  |  |
| **Sweet** |  |  |  |  |  |  |  |  |  |  |
| Lysine | 1.9±0.5^c^ | 3.8±1.1^b^ | 5.1±0.4^Bb^ | 6.9±0.6^Ba^ | 1.9±0.6^d^ | 4.3±0.7^c^ | 6.1±0.7^Ab^ | 8.5±0.9^Aa^ | <0.001 | 0.006 |
| Threonine | 4.5±0.5^Aa^ | 2.8±0.3^b^ | 4.7±0.5^Aa^ | 4.5±1.0^Ba^ | 1.0±0.4^Bc^ | 2.9±0.6^b^ | 3.8±0.3^Bb^ | 7.7±1.3^Aa^ | <0.001 | 0.040 |
| Asparigine | 1.0±0.3^Ac^ | 0.8±0.1^Bc^ | 2.9±0.4^Aa^ | 2.0±0.3^Bb^ | 0.5±0.1^Bc^ | 1.2±0.3^Ab^ | 1.3±0.1^Bb^ | 3.7±0.3^Aa^ | <0.001 | 0.021 |
| Arg/Glyc* | 113.2±6.2^a^ | 104.4±4.3^Bb^ | 95.6±2.5^Bbc^ | 95.2±2.9^Bc^ | 126.7±9.7^a^ | 125.8±6.1^Aab^ | 114.6±5.8^Ab^ | 105.8±3.9^Ac^ | <0.001 | <0.001 |
| Alanine | 18.5±1.4^Aa^ | 14.6±1.2^b^ | 11.3±1.2^Bc^ | 12.1±0.9^Bbc^ | 12.6±1.2^Bc^ | 15.4±0.9^a^ | 13.7±0.8^Abc^ | 15.1±0.8^Aa^ | <0.001 | <0.001 |
| Serine | 3.0±0.3^Ac^ | 6.7±0.4^Ab^ | 15.0±1.7^Aa^ | 15.1±1.4^a^ | 1.3±0.2^Bd^ | 4.2±1.1^Bc^ | 10.3±0.9^Bb^ | 15.1±4.3^a^ | <0.001 | 0.033 |
| *ΣSweet* | 141.0±6.7 | 133.1±2.8^B^ | 134.5±3.4^B^ | 135.7±4.9^B^ | 143.9±10.3 | 153.8±5.5^A^ | 149.7±8.1^A^ | 155.1±5.1^A^ | 0.735 | <0.001 |
|  |  |  |  |  |  |  |  |  |  |  |
| **Umami/Sour** |  |  |  |  |  |  |  |  |  |  |
| Glutamic acid | 9.0±1.0^A^ | 8.4±0.4^A^ | 9.0±1.1^A^ | 8.9±0.7^A^ | 6.8±0.7^B^ | 6.7±0.3^B^ | 6.4±0.7^B^ | 6.3±0.4^B^ | 0.104 | <0.001 |
| Aspartic acid | 0.3±0.1^Ba^ | 0.2±0.1^b^ | 0.2±0.1^Aab^ | 0.3±0.1^ab^ | 0.6±0.2^Aa^ | 0.3±0.1^b^ | 0.4±0.1^Bb^ | 0.4±0.1^b^ | <0.001 | <0.001 |
| *ΣUmami/Sour* | 9.3±1.0^A^ | 8.6±0.5^A^ | 9.2±1.2^A^ | 9.2±0.8^A^ | 7.4±0.8^B^ | 7.0±0.4^B^ | 6.8±0.8^B^ | 6.7±0.4^B^ | 0.108 | <0.001 |
|  |  |  |  |  |  |  |  |  |  |  |
| **Others** |  |  |  |  |  |  |  |  |  |  |
| Glutamine | 3.1±0.6^c^ | 3.8±0.6^c^ | 7.5±1.0^b^ | 11.6±0.8^a^ | 3.4±0.2^d^ | 7.8±0.7^b^ | 10.4±1.0^a^ | 5.0±0.8^c^ | <0.001 | 0.162 |
| ***ΣFAA*** | 183.1±9.9^bc^ | 176.8±7.3^c^ | 204.9±5.2^ab^ | 210.6±5.8^a^ | 163.1±10.1^b^ | 196.6±5.2^a^ | 204.7±21.6^a^ | 218.1±8.5^a^ | <0.001 | 0.452 |

*Arginine/Glycine could not be separated

**General linear model (GLM) analysis of variance with feeding time and species as fixed factors; where significant differences were detected (P<0.05), a Tukey's pairwise comparison or a t-test were applied.

P_FT_ and P_SP_ are the significant levels (GLM) for the effects of feeding time and species, respectively.

^a–d^Different superscript lowercase letters within each species and each FAA in the same row indicate significant differences (P<0.05) throughout feeding time.

^A-B^Different superscript uppercase letters within each feeding time and FAA in the same row indicate significant differences (P<0.05) between the species at the same feeding time.
